# Tau oligomerization induces nuclear lamina invagination and chromatin remodeling in Alzheimer’s disease

**DOI:** 10.1007/s00401-026-03018-1

**Published:** 2026-04-22

**Authors:** Shuo Yuan, Nicholas Essepian, Rebecca Roberts, Eliana Sherman, Weronika M. Gniadzik, Qingbo Wang, Alev Erisir, Lulu Jiang

**Affiliations:** 1 Department of Neuroscience, University of Virginia School of Medicine, Charlottesville, VA 22908 USA; 2 Center for Brain Immunology and Glia (BIG), University of Virginia School of Medicine, Charlottesville, VA 22908 USA; 3 Department of Psychology, University of Virginia, Charlottesville, VA 22904 USA; 4 Neuroscience Graduate Program, University of Virginia, Charlottesville, VA USA

**Keywords:** Alzheimer’s disease, Tauopathy, Optogenetics, Oligomerization, Nuclear lamina, Nuclear membrane

## Abstract

The aggregation of the microtubule-associated protein tau into oligomeric complexes is strongly correlated with the onset and progression of neurodegeneration in Alzheimer’s disease (AD). Increasing evidence implicates nuclear membrane disruption in AD and related tauopathies; however, whether this is a cause or consequence of neurodegeneration remains unresolved. Here, we show that nuclear lamina disruption emerges at the early Braak stages, coinciding with the initial formation of pathological tau aggregates in post-mortem AD brain tissue. Using the tauopathy mouse model (P301S PS19), we demonstrate that oligomeric tau (oTau) directly binds to the Lamin B Receptor (LBR), inducing nuclear envelope invaginations as revealed by electron microscopy. These structural alterations are accompanied by chromatin remodeling and gene expression dysregulation. To dissect the underlying mechanism, we employed a light-inducible OptoTau system (4R1N Tau::mCherry::Cry2Olig) in human iPSC-derived neurons, enabling real-time visualization of tau aggregation dynamics. This system revealed selective recruitment of oTau to the nuclear envelope and direct interactions with LBR and Lamin B2, leading to nuclear deformation and activation of the protein translational stress response. Together, these findings identify nuclear membrane disruption as an early and potentially causative event in tau-mediated neurodegeneration, establishing a mechanistic link between tau oligomerization, nuclear stress, and chromatin remodeling. Targeting nuclear destabilization may offer new therapeutic avenues for mitigating AD pathogenesis.

**Supplementary Information:**

The online version contains supplementary material available at https://doi.org/10.1007/s00401-026-03018-1.

## Background

Alzheimer’s disease (AD) is a progressive and debilitating neurocognitive disorder characterized by pathological β-amyloid (Aβ)-containing extracellular plaques and tau-containing intracellular neurofibrillary tangles [40], at the same time manifested by axonal withdrawal, inflammation, and accelerated neuronal degeneration [1, 27, 64]. However, whether neurodegeneration was directly caused by these pathological hallmarks and the underlying mechanism are unclear [60]. Under normal conditions, tau promotes structural integrity along microtubules; however, in AD, hyperphosphorylation of tau leads to detachment, mislocalization, and aggregation into insoluble fibrils [13, 55]. While a significant body of literature describes the biochemical alterations of tau progression, its downstream pathophysiological effects are still largely unknown [50]. Tau is typically confined to axons through tightly regulated processes, but in tauopathies, such as AD and Frontotemporal Dementia (FTD), mislocalization leads to toxic aggregation and neuronal dysfunction [50, 66]. Recent studies indicate tau oligomeric species (oTau), transitional conformations between monomers and fibrils, are the most toxic drivers of disease progression [14, 33, 67]. These highly mobile and volatile tau species appear as early players in AD pathogenesis and are prone to nonspecific protein interactions and prion-like propagation [14, 22, 58]. Tau’s propensity for aberrant interactions disrupts essential cellular processes, including proteostasis and synaptic function, potentiating neuroinflammation and neurodegeneration [9, 16, 68].

Accumulating evidence indicates that tauopathy progression is accompanied by nuclear membrane disruption [20, 34, 62], however, exact mechanisms are still a subject of debate. Implications of nuclear disturbance include pathological tau localization to the nuclear membrane, disruption of the nuclear pore complex and defects in nucleocytoplasmic transport [3, 16, 20]. The nuclear membrane plays a crucial role in maintaining genomic stability by regulating chromatin organization, transcriptional activity, and nucleocytoplasmic trafficking [29], so disruptions can lead to widespread alterations in gene expression, potentially exacerbating neurodegenerative processes [12]. Furthermore, recent studies indicate that pathogenic tau contributes to lamina destabilization, leading to nuclear invaginations and blebbing [20, 51, 53]. The latest work from our lab indicates that oligomeric tau binds directly to Lamin B2 and Lamin B receptor proteins, which renders the proteins insoluble and delocalized [35]. The nuclear lamina in nonpathological neurons is crucial to the inner nuclear membrane structure and assists in chromatin organization and mediation of the nucleocytoplasmic interface [10, 52, 71]. Interestingly, aggregate disruption of the lamina nucleoskeleton is not confined to AD pathology, as similar findings are demonstrated in other tauopathies, such as FTD, where tau aggregates recruit microtubules to nuclear deformities [53]. Therefore, it is likely that a common mechanism and mediator facilitates the binding of aggregated tau to the nuclear membrane and its entry into the nuclear compartment of affected neurons.

To address how pathological tau translocates to the nuclear membrane and contributes to nuclear disruption in AD, we examined the association between tau aggregation and nuclear lamina disruption across the Braak stages of AD. To determine whether nuclear lamina disruption is a characteristic feature of tauopathy, we analyzed early and late stages of tau aggregation in the brains of aging transgenic mice expressing human P301S (PS19) MAPT tauopathy mice, compared with age-matched controls. We further examined tau aggregation dynamics in human iPSC-derived neurons to assess whether nuclear lamina invagination and presence of pathogenic tau in the nucleus are specifically driven by the early and most toxic oligomeric forms of tau. Our findings showed that in AD patients spanning Braak stages I-VI, nuclear lamina disruption and the loss of Lamin B and its receptors strongly correlated with the emergence of early tau aggregation in intermediate-stage cases and became more severe in the late stages of the disease. Similar patterns were observed in PS19 mouse brains at 5 and 9 months of age, where pathological tau accumulation at the nuclear membrane closely correlated with the extent of lamina disruption. Electron microscopy further revealed that nuclear invagination and chromatin decompaction occur early and progressively worsen over time under tauopathy conditions. In neuronal cell culture, we used the genetically engineered construct 4R1N Tau::mCherry::Cry2Olig system to induce tau oligomerization upon exposure of 488λ blue light [34]. We find that tau granules under active oligomerization aggregate towards the nuclear membrane and induce significant intrusions into the nuclear space. Collectively, our findings demonstrate that tau oligomers exert nucleo-toxic effects that precede and potentiate neuronal dysfunction in AD.

## Materials and methods

### Human post-mortem brain tissue

Anonymous human brain tissue used in this project was obtained from the Goizuetta Alzheimer’s Disease Center and was collected in accordance with IRB protocols of Emory University. Human anterior prefrontal cortex (Brodmann area 10) was used for the immunohistochemical and immunofluorescence labeling in the current study. The samples were de-identified and are described in Supplementary Table S1. The tissue was fixed in periodate-lysine-paraformaldehyde (PLP) fixative for 2 h, followed by overnight incubation in 30% sucrose, after which tissue sections were cut at a thickness of 30 μm. All studies included both sexes, and results were integrated by covariate analysis, as described in Supplementary Table S1.

### Animals

All research was performed with approval from the University of Virginia, School of Medicine. The use of animals was approved by the University of Virginia Institutional and Animal Care and Use Committee. All animals were housed in IACUC-approved vivariums at UVA School of Medicine. The breeders C57BL/6 (Strain #:000664; RRID:IMSR_JAX:000664) and PS19 mice overexpressing human P301S Tau (B6;C3-Tg(Prnp-MAPT^∗^P301S)PS19Vle/J, Strain #:008169) were purchased from Jackson Laboratories. Male PS19 P301S tau^+/−^ and female C57BL/6 mice were used as breeding pairs and the F1 generation of P301S tau^+/−^(PS19) and P301S tau^−/−^(wild-type) were used for the experiment. Littermates of the same sex were randomly assigned to experimental groups. Mice were sacrificed for the experiment at the age of 3, 6, and 9 months old, respectively.

### Immunofluorescence (IF) labeling of fixed brain tissues

Fixed post-mortem human brain tissues and dissected mouse brain tissues were sectioned at 30 µm and stored at 4 °C in PBS containing 0.01% sodium azide. For staining, individual sections were transferred into 24-well plates (one section per well). Sections were washed in PBS for 10 min and permeabilized in 0.25% Triton X-100 in PBS (PBST) for 15-30 min. Blocking was performed in PBST containing 5% BSA and 5% normal goat serum for 1 h at room temperature. Sections were then incubated with primary antibodies diluted in 5% BSA/PBST, sealed with Parafilm to prevent evaporation, and incubated overnight at 4 °C. During primary antibody incubation, sections were photobleached using a 100-W LED lamp to reduce autofluorescence background.

The next day, sections were washed 3 times in PBST (10 min each) and incubated with secondary antibodies (1:800 in 5% BSA/PBST) for 2 h at room temperature. All subsequent steps were performed with plates covered in foil to protect samples from light. After secondary antibody incubation, sections were washed once in PBST (10 min) and then incubated with DAPI (1:5,000 dilution from a 5 mg/mL stock) for 15 min. Sections were washed twice in PBST followed by a final rinse in PBS before mounting. Then, they were mounted onto glass slides with ProLong Gold Antifade mounting medium (50 µL per slide) and covered with 12-mm glass coverslips. Slides were placed in a light-protected slide box and allowed to cure overnight at room temperature in a fume hood, then stored long-term at 4 °C.

### Immunohistochemistry single stain of fixed tissues with DAB (3,3’-diaminobenzidine)

Brain tissue  sections (30 µm) from fixed post-mortem human brain and dissected mouse brain were stored at 4 °C in PBS containing 0.01% sodium azide. For staining, sections were washed once in PBS for 10 min and incubated in 1% H_2_O_2_ diluted in distilled water for 15 min to quench endogenous peroxidase activity. Sections were rinsed twice in PBS (15 min each) and blocked for 30 min in PBS containing 0.4% Triton X-100, 1% BSA, and 4% normal goat serum (NGS). They were then incubated overnight at 4 °C with primary antibodies diluted in antibody diluent (5% BSA in 0.25% Triton X-100/PBS). Antibody sources and dilutions are listed in the Materials section.

The following day, sections were rinsed twice in PBS (15 min each) and incubated for 1 h at room temperature with biotinylated goat anti-rabbit or anti-mouse IgG (depending on the primary antibody) diluted in 0.3% Triton X-100/PBS. After two additional PBS washes (15 min each), sections were incubated with Avidin–Biotin Complex (ABC) solution (0.88% avidin and 0.88% biotin diluted in 0.3% Triton X-100/PBS) prepared 30 min prior to use. Following ABC incubation, sections were washed 3 times in PBS (10 min each). DAB solution was prepared fresh by dissolving one tablet of DAB and one tablet of urea (Sigma-Aldrich, D4193, USA) in 5 mL of distilled water. Sections were developed individually in DAB solution for 1-3 min until a medium-brown reaction product was observed, then immediately transferred to PBS. Sections were washed twice in PBS for 5 min each and mounted onto glass slides. Slides were dried overnight at 37 °C.

The next day, slides were dehydrated in graded ethanol (70%, 85%, 95%, and 100% for 2 min each, followed by 100% for 5 min). Slides were cleared twice in xylene (5 min), then coverslipped using 50 µL of Permount Mounting Medium (Fisher Scientific, SP15-500, USA). Slides were dried overnight at room temperature in a fume hood and stored long-term at 4 °C.

### Sample embedding for electron microscopy

The mice tissue intended for  electron microscopy will be fixed with mixed aldehydes (4% paraformaldehyde, 0.5% glutaraldehyde) via transcardial perfusion following the room-temperature flush of capillaries with Tyrode’s solution. Brains are fixed in the same fixatives overnight and vibratome-sectioned coronally at 50 µm thickness. Sections containing the hippocampus are treated in 1%OsO_4_ for 1 h, and in 4% uranyl acetate in 70% ethanol overnight, followed by 70%, 90%, and 100% ethanol dilutions, 5 min each, EM grade acetone (EMS-Diasum) twice for 10 min. Resin infiltration is achieved by overnight incubations first in a 50% EPON 816 (EMS) in acetone, followed by 100% resin. Sections are then flat embedded in between two pieces of Aclar and resin is left to polymerize in a 37 °C oven for two days. Regions of interest are subsequently excised and re-embedded at the bottom of Beem capsules. Trapezoids containing CA1 are cut on an ultramicrotome (Leica Ultracut 7). Ultrathin sections are examined on a JEOL 1010 electron microscope that is equipped with a 16mpixel CCD camera (SIA). Hippocampal cell somata are imaged at 4096 × 4096-pixel size.

### Immuno-electron microscopy

Free-floating vibratome sections containing the hippocampus from 9-month-old PS19 and WT mice were processed for pre-embedding immunogold labeling with silver enhancement. Sections were permeabilized in 0.05% Triton X-100 in PBS for 30 min and washed in PBS (3 times, 10 min). Low-molecular-weight blocking was performed in 0.05 M glycine in PBS for 15 min, followed by high-molecular-weight blocking in 5% BSA, 0.1% CWFS gelatin, and 5% normal goat serum in PBS for 30 min. After a brief wash in incubation buffer (IB; 0.01 M PBS containing 0.1–0.2% AURION BSA-c and 15 mM NaN_3_), sections were incubated with the primary antibody MC1 diluted in IB for 1 h at room temperature or overnight at 4 °C.

Following washes in IB (3 times, 5 min), sections were incubated overnight at 4 °C with Ultra Small ImmunoGold goat anti-mouse IgG secondary antibody (Aurion, Ultra Small ImmunoGold Reagents, gold particle diameter < 0.8 nm; Supplier: Aurion; Cat# SKU 25101). These subnanometer gold conjugates contain 60-80 µg of IgG/mL and are supplied in PBS with 1% BSA and 15 mM NaN_3_. Due to their reduced steric hindrance and minimal effect on antibody behavior, these ultrasmall gold particles are optimized for sensitive labeling and are visualized following silver enhancement. After incubation, sections were washed in IB (6 times, 5 min) and PBS (3 times, 5 min), then post-fixed in 2% glutaraldehyde in 0.1 M phosphate buffer for 30 min. Sections were washed in phosphate buffer (2 times, 5 min) and distilled water (5 times, 2 min) before silver enhancement. Silver enhancement was performed using the Aurion R-Gent SE-EM kit following pre-conditioning in Enhancement Conditioning Solution (10 times diluted; 4 times, 5 min), then incubating the tissue in the enhancer/developer mixture for 1-3 h on a shaker. Sections were washed in ECS (4 times, 10 min), washed in 0.1 M phosphate buffer (2 times, 10 min), and treated with 0.5% osmium tetroxide for 15 min. After osmication, sections were dehydrated through a graded ethanol series, transitioned through acetone, infiltrated with resin, and flat-embedded. Ultrathin sections were cut and examined using a transmission electron microscope.

### Human iPSC induced neurons cell culture

All cell lines were cultured under standard conditions (37 °C, 5% CO_2_ atmosphere). Cell viability was routinely assessed using the RWD Cellometer system with 0.4% trypan blue staining (Thermo Fisher), with duplicate measurements performed for each sample.

Human-induced pluripotent stem cells (iPSCs) were sourced from the JAX iPSC Collection (KOLF2.1 J line, product code #JIPSC001000). Neural progenitor cell (NPC) differentiation was initiated using STEMdiff^™^ Forebrain Neuron Differentiation Media (Stem Cell Technologies, #08600, USA) in Matrigel^®^-coated plates (Corning^®^, #354,277, USA). NPCs were maintained in serum-free STEMdiff^™^ Neural Progenitor Medium 2 (#08560) with the following parameters:

Seeding density: 50,000 cells/cm^2^; Passage criteria: 90% confluency using Accutase^™^ dissociation (#07920); Medium refreshment: Complete replacement every 2-3 days; Cryopreservation: Early-passage cells (*P* < 3) in neural medium supplemented with 10% DMSO; Experimental use restriction: ≤ P6 passages.

To promote terminal differentiation, passage-competent NPCs were transduced with NEUROG2 lentiviral particles (GeneCopoeia, #LPP-T7381-Lv105-A00-S, USA) at a multiplicity of infection (MOI) of 3, generating human iPSC-derived neuronal cells (iNeurons). The complete medium exchange was conducted 24 h post-transduction to remove residual viral particles.

### OptoTau lentivirus transduction in iNeurons

Four 75-cm^2^ flasks were prepared and designated as mCherry 0_min, mCherry 60_min, OptoTau 0_min, and OptoTau 60_min. Each flask was pre-coated for 2 h with 8 mL DMEM/F-12 medium (STEMdiff^™^, #36,254, USA) supplemented with 40 µL Matrigel (Corning^®^, #354,277, USA). After coating, cells were dissociated, resuspended in Neural Progenitor Medium (STEMdiff^™^, #05834, USA) at 6 × 10^5^ cells/mL, and plated into the prepared flasks. Once cells adhered, lentiviral transduction was initiated by adding 4 µL/mL mCherry lentivirus to the mCherry groups or 4 µL/mL OptoTau lentivirus to the OptoTau groups. After 48 h, a second round of lentiviral treatment was performed, and 1 µL/mL NEUROG2 lentivirus (GeneCopoeia, CLP-HPRM50462, USA) was added. At this time, the culture medium was replaced with Forebrain Neuron Maturation Medium (STEMdiff^™^, #08605, USA). Cultures were maintained by refreshing medium every 2-3 days.

For light-induced OptoTau activation, the mCherry 60 min and OptoTau 60 min groups were exposed to 488-nm blue light for a total of 60 min. A mounted LED system delivering 2.5 nW/mm^2^ was used to achieve the activation threshold for Cry2Olig. To minimize phototoxicity while effectively inducing OptoTau oligomerization, neurons were exposed to pulsed illumination consisting of 20 min of light followed by 20 min of darkness, repeated for three cycles (total 60 min of light exposure time). Following light stimulation, cells were harvested for lysate collection or fixed and stained for nuclear membrane markers.

### Live cell staining with NucSpot Live 488

Human iPSC-derived neuron cultures and OptoTau transductions were repeated in 35 mm glass-bottom dishes without prolonged blue-light exposure. One hour before live-cell imaging, neurons were stained with NucSpot Live 488 (Biotium, #40,081), a low-toxicity nuclear DNA dye suitable for live imaging. NucSpot (1000 × stock) was diluted to 1 × in neuronal forebrain maturation medium and applied to cells for 15-20 min at 37 °C. The staining solution included 25 µM verapamil to reduce nuclear dye efflux. After incubation, the staining medium was replaced with fresh forebrain maturation medium, and cells were equilibrated for 20-30 min to allow optimal pH under 5% CO_2_. To maintain stable pH during live imaging, the medium was supplemented with 25 mM HEPES buffer.

### Immunofluorescence (IF) labeling of fixed iPSC-derived neurons

In 24 well plates of glass bottom, cells were fixed with 0.5 mL 4% PFA/PBS for 10 min and washed 3 times in PBS for 5 min. Cells were permeabilized in 0.5 mL PBS/0.1% Triton X-100 (PBST) for 15-30 min and blocked in 0.5 mL of 5% BSA-5% goat serum in PBST for 1 h. Cells were then incubated in primary antibody diluted in 5% BSA/PBST overnight at 4 °C. Plates were wrapped in Parafilm and photobleached by the LED lamp. The next day, the cells were washed 3 times in PBS-T, 10 min each before being incubated in secondary antibody diluted 1:800 in 5% BSA/PBST, for 2 h at room temperature. In all remaining steps, cells were covered in foil to prevent ambient light photobleaching. For antibody information and dilutions, view the materials section. After the secondary antibody, cells were washed one time in PBS-T for 10 min and incubated in DAPI diluted 1:10,000 in PBST (5 mg/ml stock solution) for 15 min after the first wash. Then cells were washed twice with PBST, and once with PBS, 10 min each. The Prolong Gold Antifade mounting media was dropped in the middle of each well and covered with 12 mm coverslips. Plates were covered from light and dried in the fume hood overnight before long-term storage at 4℃.

### BCA protein assay

BSA standards were prepared using the Pierce^™^ BCA Protein Assay Kit (#23,227, Thermo Fisher, USA). The BSA stock was diluted into a series of concentrations in separate clean tubes to generate a standard curve. BCA Working Reagent was prepared by mixing Reagents A and B in a 50:1 ratio. In a 96-well plate, 12.5 µL of each sample or standard was combined with 100 µL of Working Reagent. Plates were covered with aluminum foil and incubated at 37 °C for 30 min. Absorbance was measured at 562 nm, and protein concentrations were determined using the standard curve. For sample preparation, lysates were mixed with 4 × Bolt^™^ LDS Sample Buffer (#B0007, Thermo Fisher, USA), 10 × reducing reagent (#B0009, Thermo Fisher, USA), and PBS, then heated at 100 °C for 10 min to fully denature the proteins.

### Western blot

Protein samples were loaded onto Bolt^™^ Bis-Tris Plus Mini Protein Gels (Thermo Fisher, #NW04127BOX, USA). PageRuler^™^ Plus Prestained Protein Ladder (Thermo Fisher, #26,619, USA) was included to monitor electrophoresis and determine protein molecular weights. Electrophoresis was performed using a Gel Runner Tank (Thermo Fisher, #B4478641, USA) with running buffer (Invitrogen^™^, #B000202, USA), starting at 80 V for 10 min and then 120 V for approximately 1 h. Proteins were transferred to nitrocellulose membranes using transfer buffer (Invitrogen^™^, #BT0006, USA) at 20 V for 120 min. Membranes were blocked with 5% skim milk in PBST (bioworld, #30,620,074-1, USA) for 60 min at room temperature with gentle agitation.

Membranes were incubated overnight at 4 °C with primary antibodies diluted according to manufacturer’s instructions. The following day, membranes were washed 3 times with PBST for 10 min each. Membranes were then incubated with the appropriate secondary antibodies for 1 h at room temperature with gentle shaking, followed by three additional PBST washes (10 min each). Protein detection was performed using a gel imager (Bio-Rad), and bands were quantified using ImageJ (Fiji) software.

### Immunoprecipitation assay

Immunoprecipitation was performed using the ChromoTek RFP-Trap^®^ Agarose kit (Cat. No. RTA, Proteintech, USA) for mCherry co-IP and Pierce^™^ Protein A/G Magnetic Beads (Cat. No. 88802, Thermo Fisher Scientific, USA) for TOMA2 co-IP experiments. Beads were resuspended by gentle pipetting or inversion, and 25 µL of bead slurry was transferred into a 1.5-mL microcentrifuge tube. Beads were washed with 500 µL of ice-cold PBS and pelleted either by centrifugation at 2500 × g for 5 min or using a magnetic rack at 4 °C. For the mCherry co-IP, clarified and diluted lysates were added to the equilibrated beads after removing the supernatant, followed by incubation with end-over-end rotation for 1 h at 4 °C. For the TOMA2 co-IP, 5 µg of antibody was added to the lysate prior to mixing with the magnetic beads.

Beads were pelleted again at 2500 × g for 5 min at 4 °C, and the supernatant was collected for additional analysis if needed. Beads were washed 3 × with 500 µL PBST (PBS + 0.1% Tween-20), with the final wash transferred to a fresh tube to minimize carryover. After the final wash, residual buffer was removed and beads were resuspended in 80 µL of 4 × sample buffer. Samples were boiled for 5 min at 95 °C to elute bound immunocomplexes, followed by centrifugation at 2500 × g for 2 min at 4 °C. The resulting supernatant containing the immunoprecipitated proteins was analyzed by Western blot. Specific details regarding primary and secondary antibodies are provided in Supplementary Table S2.

### Quantification and statistical analysis

#### Image analysis

Fluorescence labeling intensity was measured using FIJI (ImageJ). Colocalization of tau isoforms and lamina components was analyzed using the Coloc2 plugin for FIJI. Quantification of nuclear invagination rates by ROI was done using a custom plugin “Nuclear Laminin Distribution” for FIJI provided by Paonessa F. at the Livesey Lab [53]. The plugin uses the dsDNA DAPI staining channel to map nuclear bounds and the lamina channel to map internal and peripheral lamina components. Nuclei with an internal lamina area > 0.3 of total lamin are counted as invaginated. Additional channels can be used to measure mean intensity within the nuclear bounds. For EM quantification, invagination size was measured by relative membrane length using FIJI. Relative chromatin coverage was measured using FIJI. Chromatin clump density was measured using the “connected components labeling” tool from the MorpholibJ plugin for FIJI [47]. Clumps were quantified as close pixels with an area ≥ 0.04 mm^2^ and normalized to the nucleus area.

### Statistical analysis

Statistical analyses and figures artwork were performed using GraphPad Prism version 10.00 for Windows with a two-sided α of 0.05. All group data are expressed as mean ± SEM. Column means were compared using one-way ANOVA with treatment as the independent variable. Group means were compared using two-way ANOVA using factors of genotype and fraction treatment, respectively. When ANOVA showed a significant difference, pairwise comparisons between group means were examined by Tukey’s, Dunnett’s, or uncorrected Fisher’s LSD multiple comparison test. Significance was defined when *p* < 0.05.

## Results

### Progressive loss of Lamin B receptor-dependent nuclear membrane integrity parallels early tau misfolding and precedes symptomatic AD

**Fig. 1:**
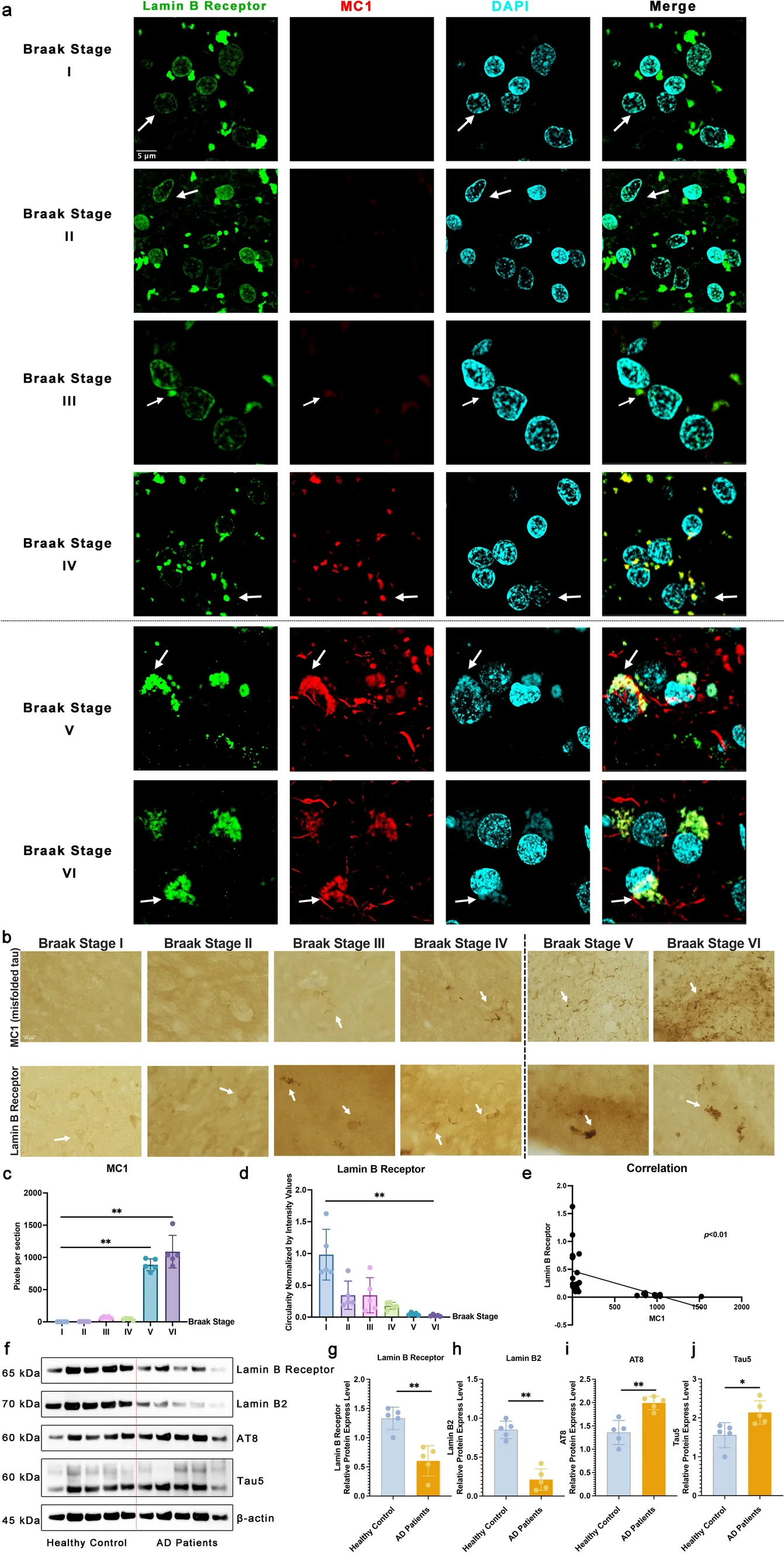
Progressive loss of Lamin B receptor-dependent nuclear membrane integrity parallels early tau misfolding and precedes symptomatic AD. **a** Representative confocal images of colocalization of MC1-positive misfolded tau with Lamin B Receptor in human post-mortem brains. (Green: Lamin B Receptor; Red: MC1; Cyan: DAPI). The scale bar = 20 µm. Arrows indicate alterations in nuclear membrane integrity and the co-localization of the lamin B receptor with MC1-positive misfolded tau in the AD brain. **b** Representative images of DAB staining for MC1 (top) and Lamin B receptor (bottom) in human post-mortem brain tissue. Arrows indicate that at Braak stage III, MC1-positive misfolded tau begins to appear in patient brains, with a marked increase in MC1-positive tau aggregates at Braak stages V and VI. Similarly, the lamin B receptor remains largely intact in early stages, while significant nuclear membrane rupture is observed at stages V and VI. **c** Quantitation of the normalized integrated density of MC1 (misfolded tau) in DAB staining by Braak stage. *n* = 5. **d** Quantitative analysis of the nuclear integrity labeled by Lamin B receptor using IHC staining with DAB by Braak stages, *n* = 5. Integrity is calculated as circular continuity of pixels within each ROI. **e** The correlation analysis of nuclear integrity labeled by Lamin B receptor with MC1-positive misfolded tau aggregation, *n* = 5. The Pearson analysis was used for experiments, r = -0.5373, *p* < 0.01. Data shown as mean ± SEM. Analysis was conducted using one-way ANOVA with Tukey’s multiple comparisons test in (**c**) and (**d**). * *p* < 0.05, ** *p* < 0.01. **f** Representative Western blot images showing decreased levels of Lamin B2 and Lamin B Receptor (LBR), along with the accumulation of AT8, and Tau5 in human brains. **g-j** Quantification of Western blot results shown in (**f**). Analysis was conducted using* t* test. * *p* < 0.05, ** *p* < 0.01

To address how early the pathological tau translocates to the nuclear membrane and contributes to nuclear disruption in AD, we examined the association between tau aggregation and nuclear lamina disruption across the Braak stages of AD in comparison to age-matched normal controls. Brain tissues were grouped by Braak stage, and immunohistological analysis was performed on Brodmann area 10 of the prefrontal cortex. We probed for misfolded tau by MC1 antibody [36] and Lamin B Receptor to track pathological tau accumulation and nuclear disruption. Our results revealed stage-dependent nuclear pathology. Asymptomatic controls (Braak I-III) exhibited continuous perinuclear Lamin B Receptor staining with minimal MC1 signal (Fig. 1a). In early tau pathology cases (Braak IV), Lamin B Receptor staining started to present as fragmented and co-localized with initial MC1 aggregates while advanced AD cases (Braak V-VI) showed complete disintegration of Lamin B Receptor staining (Fig. 1a).

Additionally, we performed 3,3′-diaminobenzidine (DAB) staining on the same Brodmann area 10 of the prefrontal cortex to confirm the co-occurrence of nuclear lamina disruption and tau misfolding during disease progression. The results consistently showed early, but mild MC1-positive misfolded tau in Braak stages III and IV and markedly increased tangle accumulation in stages V and VI (Fig. 1b, top panel). Quantification of MC1 signal revealed a sharp increase in stages V and VI compared to stages I-IV (Fig. 1c). These findings support well-established observations that smaller tau oligomers emerge early in disease progression, whereas larger tau complexes appear in late-stage AD pathology [2, 46]. Strikingly, Lamin B Receptor staining demonstrates progressive loss of nuclear integrity in tandem with early misfolded tau emergence (Fig. 1b, bottom panel). In early control Braak stages I-III, Lamin B Receptor shows high nuclear circularity, indicative of intact nuclear envelopes, whereas stage IV tissue shows the emergence of disrupted, fragmented staining patterns, reflective of compromised nuclear morphology (Fig. 1b, bottom panel). Quantification of nuclear integrity calculated as circular continuity within nuclear ROIs reveals significant decreases in LBR nuclear integrity as early as Braak stages II-III, with continued decline into later stages (Fig. 1d). Correlation analysis between MC1-positive tau pathology and Lamin B Receptor circularity confirms a significant decrease in nuclear Lamin B Receptor integrity from control to AD brain tissue, as assessed by both linear (Fig. 1e) and logarithmic regression models (Supplementary Fig. S1a). Additional analyses were performed to assess the potential contribution of age to Lamin B receptor reduction (Supplementary Fig. S1b-d). Stratification into three age groups (59-74, 75-90, > 90 years) revealed no significant differences (*p* = 0.5710). In contrast, Lamin B receptor levels were significantly reduced in AD cases compared with controls (*p* = 0.0019), and this effect remained significant after adjusting for age. No significant correlation was observed between Lamin B receptor levels and age (linear regression, *p* = 0.5710). These findings indicate that Lamin B receptor reduction is associated with disease status rather than aging. Particularly, quantified disruption emerged concurrently with mild MC1 positive tau aggregation and became compromised prior to severe fibril accumulation. These findings suggest that nuclear membrane disruption occurs in early AD pathogenesis. Initiation of nuclear disturbance by tau oligomers prior to extensive fibril accumulation supports our hypothesis that lamina interference may be an initiating factor in neuronal dysfunction. Furthermore, Western blot results in Fig. 1f-j also showed that in the brains of AD patients, the expression levels of Lamin B2 and Lamin B Receptor were significantly decreased, while the levels of AT8 positive tau phosphorylation and Tau5 positive total tau level were increased (*p* < 0.05). To further evaluate the relationship between tau pathology and nuclear membrane components, we performed correlation analyses between AT8 and Tau5 levels and Lamin B2 and Lamin B receptor levels based on Western blot quantification. As shown in Supplemental Fig. S1e-h, Lamin B2 levels were significantly negatively correlated with both AT8 and Tau5 levels, indicating that increased tau pathology is associated with reduced Lamin B2 expression. In contrast, although Lamin B receptor levels did not show statistically significant correlations with AT8 or Tau5, a consistent decreasing trend was observed with increasing tau levels. Altogether, these findings support the association between tau accumulation and nuclear lamina alterations, and emphasize the role of nuclear destabilization as an early and potentially causative pathogenic feature of AD.

### Nuclear invaginations and lamina disruption precede neurodegeneration in tauopathy mouse

**Fig. 2:**
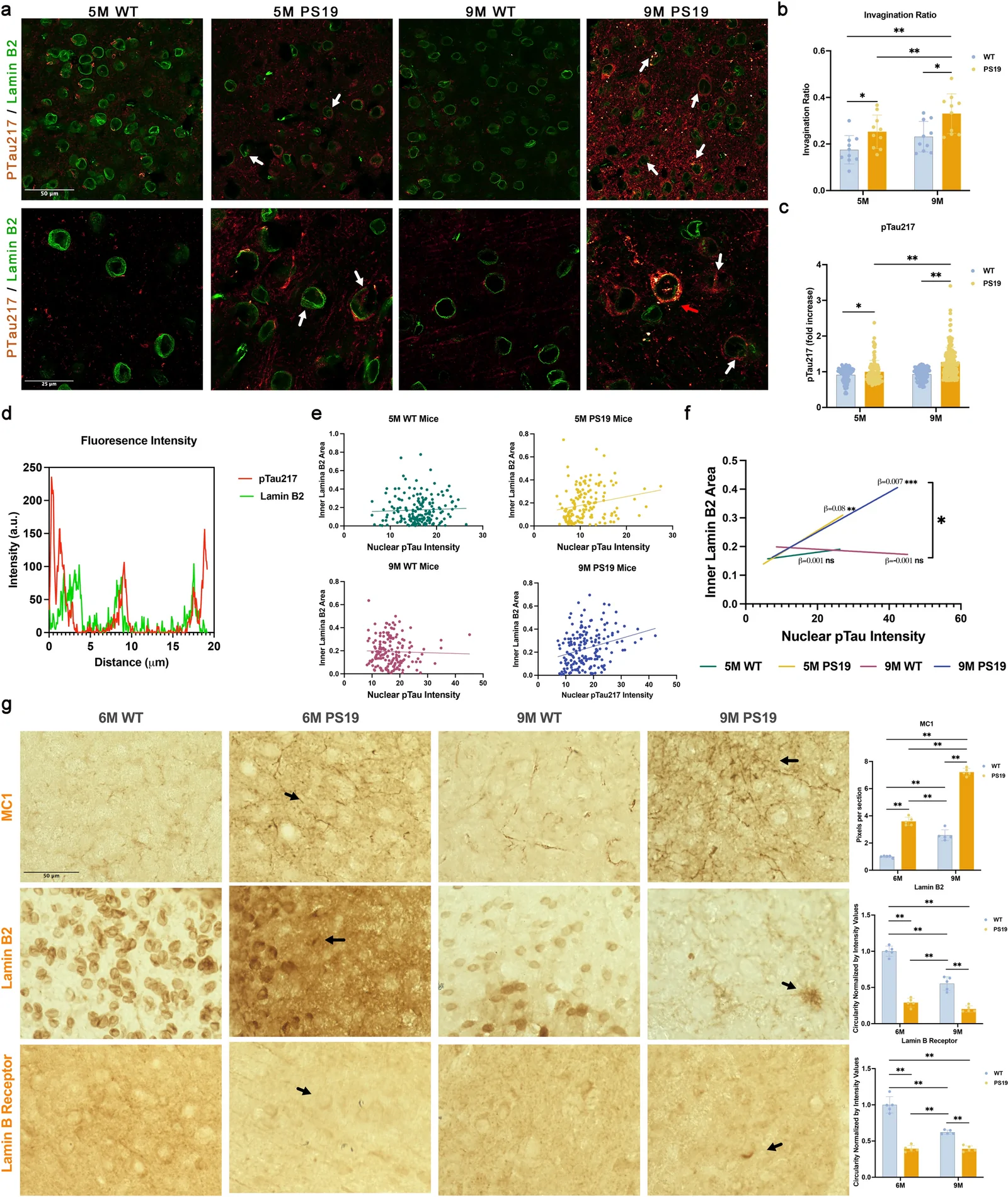
Nuclear invaginations and lamina disruption precede neurodegeneration in tauopathy mouse. **a** Representative images of postnatal month-5 and month-9 neurons from PS19 tau frontal cortex compared to C57 WT. The scale bar = 50 or 25 µm as indicated in the image. Arrows indicate areas of nuclear membrane invagination with visible folds in the nuclear lamina, showing localized accumulation of tau along these invaginations. **b** Quantitative analysis for the ratio of nuclei that present with invagination (internal Lamin B2 ratio ≥ 0.3). **c** Quantitation for the pTau217 mean fluorescence intensity accumulated in the brain cortex of 5-month and 9-month mice. Raw pTau217 intensity was normalized to cell number, followed by fold-change comparisons. **d** Representative fluorescence intensity histogram of a nuclear membrane invagination, showing the co-localization and overlapping intensity profiles of pTau217 and Lamin B2 in the neuronal cell indicated by the red arrow in the representative image in (**a**).**e** Correlation between mean pTau217 intensity within the nuclei and inner lamina proportion. **f** Correlation slopes from (**e**) plotted on a combined axis. *n* = 158 to 177 nuclei. For significantly nonzero slopes of 5 m WT, 9 m WT, 5 m PS19, 9 m PS19, *p* = 0.56, 0.68, 0.0078 (**), 0.0001 (***), respectively. For the difference in slopes, *p* = 0.012. Solid lines indicate one-way ANOVA; dashed lines indicate unpaired *t* test. **g** Representative IHC DAB staining images and quantification showing increased MC1-positive tau accumulation and decreased Lamin B2 and Lamin B Receptor levels in the brains of 6- and 9-month-old PS19 mice compared to WT controls. Arrows indicate MC-1-positive misfolded neurofibrillary tau tangles and nuclear membrane disruption labeled by lamin B2 and the lamin B receptor (LBR). Data are presented as mean ± SEM. Two-way ANOVA with Tukey’s multiple comparisons test was used for quantitative analysis in (**b**), (**c**), and (**g**). *p < 0.05, **p < 0.01, *n* = 5

To further determine whether nuclear lamina disruption is specifically caused by tau pathology rather than other complex pathogenic processes of AD, we utilized the PS19 tauopathy mice and compared them with C57BL/6 wild-type (WT) mice as age-matched controls. The PS19 mice harbor the P301S human mutation to MAPT with one N terminal insert and four microtubule binding repeats (4R1N) [24, 30, 69]. They are a well-established transgenic mouse strain presenting mature features of tau pathology and recapitulating tau-mediated neurodegeneration in AD [30, 69]. The neurofibrillary tangle-like inclusions began to appear at 6 months while neuronal loss and brain atrophy occurred after 8 months [28]. In the present study, we collect brains of WT and PS19 mice at 5 and 9 months of age, respectively (Fig. 2a).

Neurons in the cortex of 5- and 9-month-old PS19 mice exhibit higher levels of phosphorylated tau compared to the WT age-matched controls (Fig. 2a). These neurons also display a remarkable incidence of misshapen and invaginated nuclei, accompanied by the localization of pathological tau (Fig. 2a). The nuclear invagination was assessed by quantifying intranuclear Lamin B2, which serves as a parameter of nuclear folding. Nuclei with internal Lamin B2 at a ratio of 0.3 or more of total Lamin B2 qualify as invaginated. Quantification of ROI in WT and PS19 cortex reveals significant increases in incidence of nuclear invagination among 5- and 9-month PS19 mice over age-matched controls (Fig. 2b). Notably, rates of nuclear disruption are comparable between 5 m PS19 and 9 m WT, indicating nuclear invagination as an early event in disease progression and implying accelerated neuron aging through lamina disruption [43, 44].

By mapping early tau phosphorylation using pTau 217 tau antibody (recognition of the phosphorylation site at threonine 217, recognizes phosphorylated mouse tau in addition to human tau) within individual neurons in colocalization with Lamin B2, we found that phosphorylated tau was consistently elevated (Fig. 2c) and colocalized with nuclear invaginations in the brains of 5- and 9-month-old PS19 mice compared with WT controls, which is consistent with Fig. 2a. A fluorescence intensity histogram from a representative neuron revealed elevated pTau signals in close proximity to the outer lamina and a direct overlap of pTau with Lamin B2 within an internal nuclear membrane fold (Fig. 2d). Moreover, correlating nuclear pTau 217 mean intensity with internal Lamin B2 of individual nuclei reveals that only 5- and 9-month PS19 mice show significantly nonzero correlations (*p* = 0.0078, 0.0001, respectively) and are significantly greater than their WT counterparts (Fig. 2e, f). Additionally, we performed DAB staining to provide extra evidence of progressive nuclear lamina disruption associated with pathological tau accumulation. Compared to WT mice, 6- and 9-month-old PS19 mice presented significantly increased MC1-positive misfolded tau deposition (Fig. 2g). In WT mouse brains, nuclear lamin appeared as an intact, membrane-associated structures, whereas in PS19 mice, nuclear lamin appeared fragmented, indicative of nuclear membrane disruption (Fig. 2g). The quantitative data also suggested a decrease in total LaminB2 and LBR levels in the PS19 tauopathy mice. Taken together, these findings demonstrate that pathological tau directly binds to and disrupts components of the nuclear lamina. More importantly, they indicate that nuclear invagination and lamina disruption are strongly correlated with pathological tau accumulation and occur prior to overt neurodegeneration during disease progression.

### Pathological tau-bearing neurons display nuclear deformation and chromatin reorganization

**Fig. 3:**
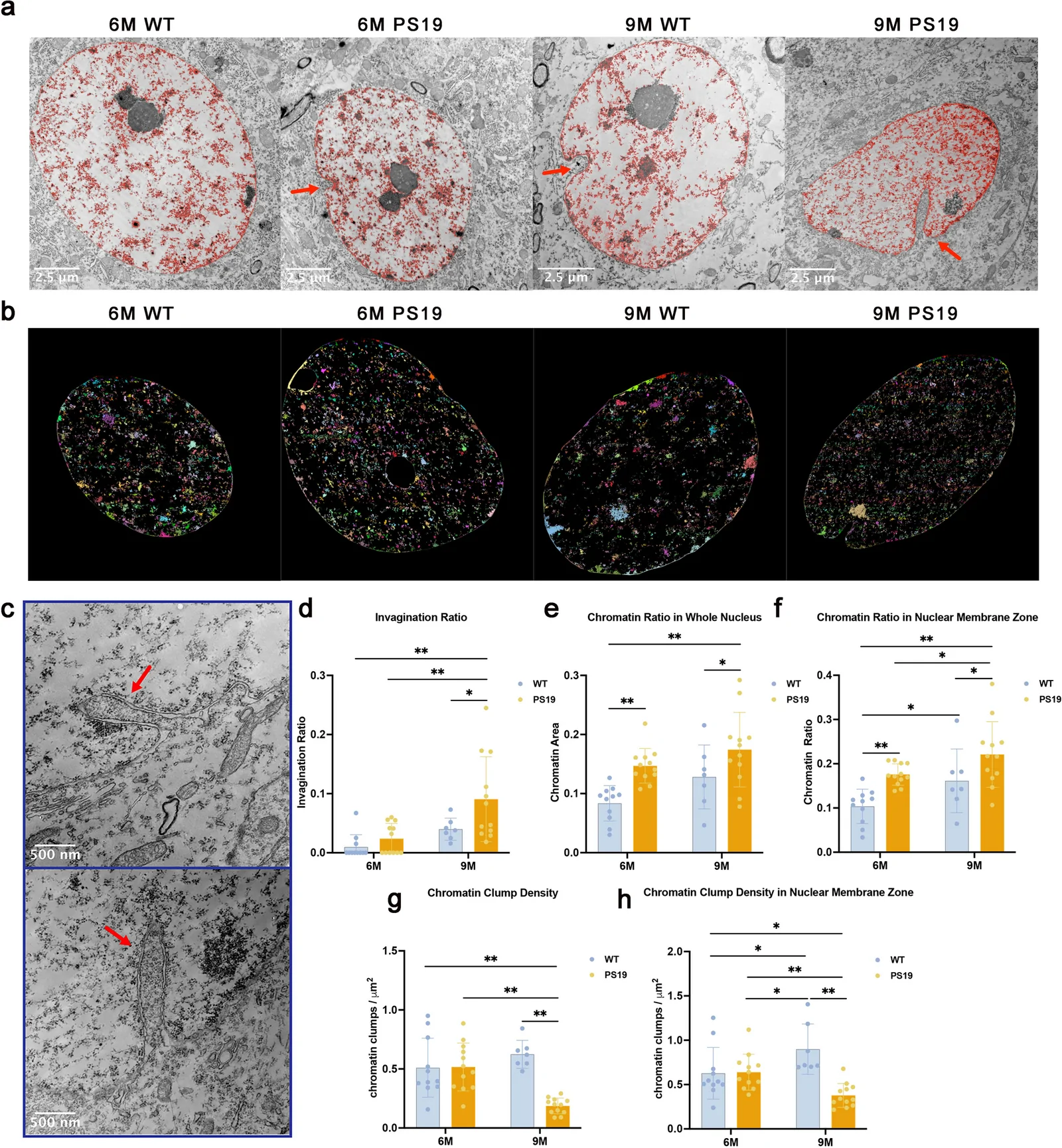
Pathological tau-bearing neurons display nuclear deformation and chromatin reorganization. **a** Representative electron microscopy (EM) images of neurons in the hippocampus CA1 at 4 k × magnification. Chromatin is outlined in red. The scale bar = 2.5 µm. **b** Representative connected components labeling of chromatin at 4 k × magnification. Components are defined as closely connected pixels. **c** Representative EM images of neurons with nuclear invaginations at 15 k × magnification. The scale bar = 500 nm. Arrows indicate areas of nuclear membrane invagination with chromatin clumps. **d** Quantitation for the ratio of invagination nuclear membrane distance relative to the perimeter of the whole nucleus. **e** Quantification for the chromatin area coverage relative to the entire nucleus excluding nucleoli across conditions. **f** Quantification for the chromatin area coverage relative to the area of the nuclear membrane zone (NMZ), the outermost 20% region of the nucleus along the inside of the nuclear membrane. **g** Quantification for the density of chromatin clumps normalized to whole nucleus area excluding the nucleoli. Clumps are chromatin components with an area ≥ 0.04 µm^2^. Lower values correspond with lower chromatin clump density. **h** Density of chromatin clumps within the NMZ normalized to the NMZ area. Error bars indicate 95% SEM. *n* = 8 to 14. The two-way ANOVA with Tukey’s multiple comparisons test was used for the quantitative analysis, *p < 0.05, **p < 0.01

Building on our previous results showing elevated rates of nuclear invagination in PS19 tau mouse brains compared to age-matched controls (Fig. 2), we continued to examine ultrastructural abnormalities in both nuclear shape and chromatin density by application of transmission electron microscopy (EM) in brain samples from the 6-month and 9-month Tau P301S PS19 mice in comparison to the normal aging of C57BL/6 WT. EM imaging of hippocampal neuron nuclei reveals emergence of nuclear envelope abnormalities including large invaginations protruding into the intranuclear space in the cortex of the PS19 tau mouse brain (Fig. 3a, b, c). Quantification revealed that nuclear invaginations were most frequent and exhibited the greatest relative depth in 9-month-old PS19 mice; however, smaller intrusions were also observed in 6-month-old PS19 mice brain (Fig. 3d). Notably, invaginations with similar incidence and size to those observed in 6-month-old PS19 mice were also present in 9-month-old WT mice, suggesting nuclear lamina folding as a feature of physiological aging, which was also reported in previous research [43, 44]. However, the increased frequency and severity of invaginations in tau mice indicate a disease-accelerated aging phenotype.

Given the importance of lamina stability in maintaining genomic integrity during aging [15, 43, 44], we investigated whether lamina structural aberrations coincide with broad changes in chromatin organization. Previous studies have indicated that tauopathy progression is linked with genomic reorganization, particularly chromatin relaxation marked by decreased heterochromatin content [21, 23]. Using chromatin area coverage and connected components labeling analysis, we assessed chromatin distribution within the nuclei (Fig. 3b). Our results demonstrated that neurons from 6 and 9-month PS19 mice showed global chromatin loosening compared with age-matched controls (Fig. 3e, g). This was reflected in increased chromatin coverage across the nucleus (Fig. 3e) and decreased chromatin clump density in 9-month tau mice, revealing that tauopathy promotes heterochromatin loss and nuclear decompaction, particularly in neurons presenting with greatest lamina disruption (Fig. 3g). To further localize the effects of Lamin B2 disruption, we defined a nuclear membrane zone (NMZ) as the outermost 20% of the nucleus to capture changes in perinuclear chromatin, which is rich in lamina-associated domains (LADs) [44, 65]. Within the NMZ, cortical neurons in the PS19 mouse brain exhibited increased chromatin coverage compared with age-matched controls (Fig. 3f). The NMZ also showed greater relative decreases in chromatin density in the outer nucleus (Fig. 3h), further demonstrating increased euchromatin dispersal in response to nuclear invagination related stress. Since this region is highly enriched in LADs, it is plausible that lamina destabilization would impair genome anchoring. In addition, we performed immunoelectron microscopy and found that MC1-positive misfolded tau was extremely low in 9-month-old WT mice, whereas PS19 mice exhibited markedly elevated levels of MC1, which were predominantly localized to the invaginated regions of the nuclear membrane (Supplemental Fig. S2).

Collectively, these findings provide comprehensive evidence that tau pathology induces ultrastructural changes in nuclear membrane morphology, accompanied by global chromatin reorganization. Decreased heterochromatin content, particularly in the NMZ, further implicates lamina dysfunction in the degradation of genome organization. Altered genetic architecture may underlie aberrant transcription patterns observed in AD that mediate accelerated degeneration [23, 39, 59]. Finally, these data reinforce the concept of tau-driven acceleration of neuronal aging and highlight chromatin as one of the key targets of tau toxicity.

### Tau oligomerization in iPSC-derived neurons induces tau accumulation and binding at the nuclear membrane, leading to nuclear invagination

**Fig. 4:**
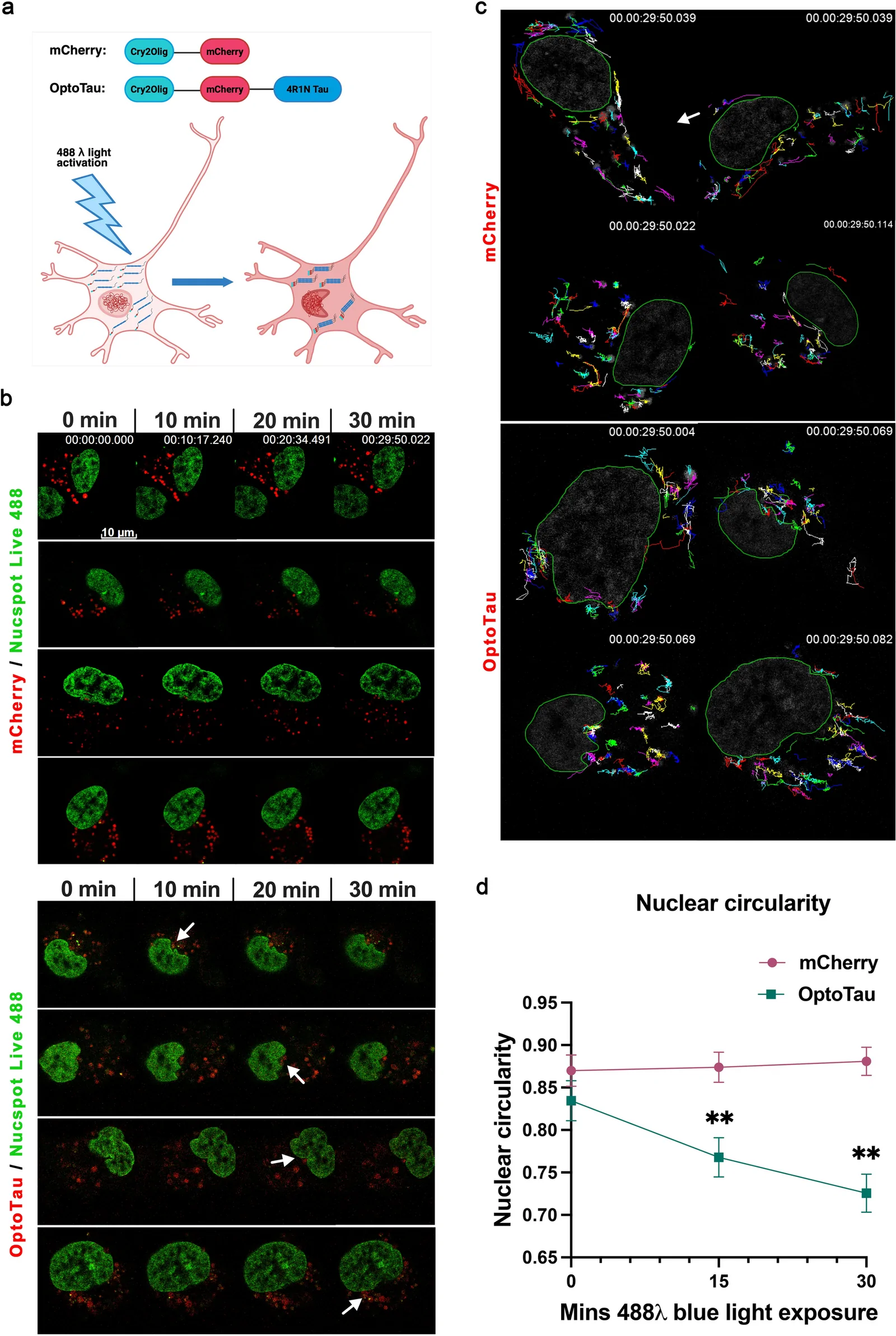
Tau oligomerization in iPSC-derived neurons induces tau accumulation and binding at the nuclear membrane, leading to nuclear invagination. **a** Schematic illustrating OptoTau and mCherry vector design and mechanism of light-inducible protein aggregation. **b** Representative time-lapse images of mCherry and OptoTau expressing neurons stained with Nucspot Live 488 at 0, 10, 20, and 30 min of blue light exposure. The scale bar = 10 µm. **c** Closeup representative images of neuronal nuclei at 30-min timepoint overlaid with particle tracking lines following mCherry or OptoTau fluorescent granules over the time course. Green outline denotes the nucleus at 30 min. **d** Nucleus circularity at 0, 15, and 30 min of blue light exposure. Data shown as Mean ± SEM. *N* = 9-10. The two-way ANOVA with Tukey’s multiple comparisons test was used for experiments, **p < 0.01, ***p < 0.001

To further determine whether nuclear invagination is induced by early tau aggregation species (tau oligomers) and to track the dynamic interaction of misfolded tau with the nuclear membrane, we examined tau oligomerization in real time using iPSC-derived neurons transduced with either 4R1N Tau::mCherry::Cry2Olig (OptoTau) or mCherry::Cry2Olig (mCherry) control constructs [34]. These systems use fluorescent mCherry linked with the protein Cry2Olig which dimerizes under 488λ blue light exposure (Fig. 4a), however, the mCherry control lentiviral vector has no additional linked protein while OptoTau expresses the 4R1N tau isoform, a key mediator of AD tau toxicity [6, 34]. This means that while mCherry forms temporary dimers of Cry2Olig, OptoTau facilitates prolonged aggregated granules by facilitating stable oligomerization of 4R1N tau proteins (Fig. 4a). This makes OptoTau a powerful tool for inducing and observing the spatiotemporal aggregation of tau in real time under a controlled time course (Fig. 4b). In addition to mCherry fluorescence, we used the cell membrane permeable DNA dye Nucspot Live 488 designed for live cell imaging applications to mark the nuclei.

Previous studies of AD and similar neurodegenerative conditions have found atypical protein aggregates to cause nuclear membrane abnormalities including destabilization, invagination, leakiness, and rupture [8, 16, 35, 42, 53, 62]. Consistent with established findings, 30-min blue light time courses exhibit OptoTau aggregate localization to the nucleus and nuclear misshaping (Fig. 4b, Videos S1, 2). Neurons with the mCherry control show presence and movement of fluorescent granules within the cell body but granular motility was largely random and did not localize at the nucleus (Fig. 4b). Particle tracking over 30-min confirms mCherry granules exhibit random movement patterns within cell bodies and around the nuclear envelope while OptoTau aggregate progressive lines frequently converge toward forming cavities in the nucleus and eventually cross the nuclear boundary (Fig. 4c). Further analysis at 0-, 15-, and 30-min time points reveals that although mCherry and OptoTau nuclear circularity begin at similar means of 0.87 and 0.83, respectively, OptoTau nuclear circularity decreases significantly with blue light exposure while mCherry remains stable (Fig. 4d). This finding aligns with the observation that fluorescent OptoTau aggregates induce concavities in the nuclear envelope during blue light activation (Fig. 4b). More importantly, this result confirms that nuclear abnormalities are caused by active oligomerization of 4R1N tau, rather than by the mCherry::Cry2Olig control.

### OptoTau-induced oligomerization leads to sustained accumulation of pathological tau that membranes and penetrates the nucleus in iPSC-derived neurons

**Fig. 5:**
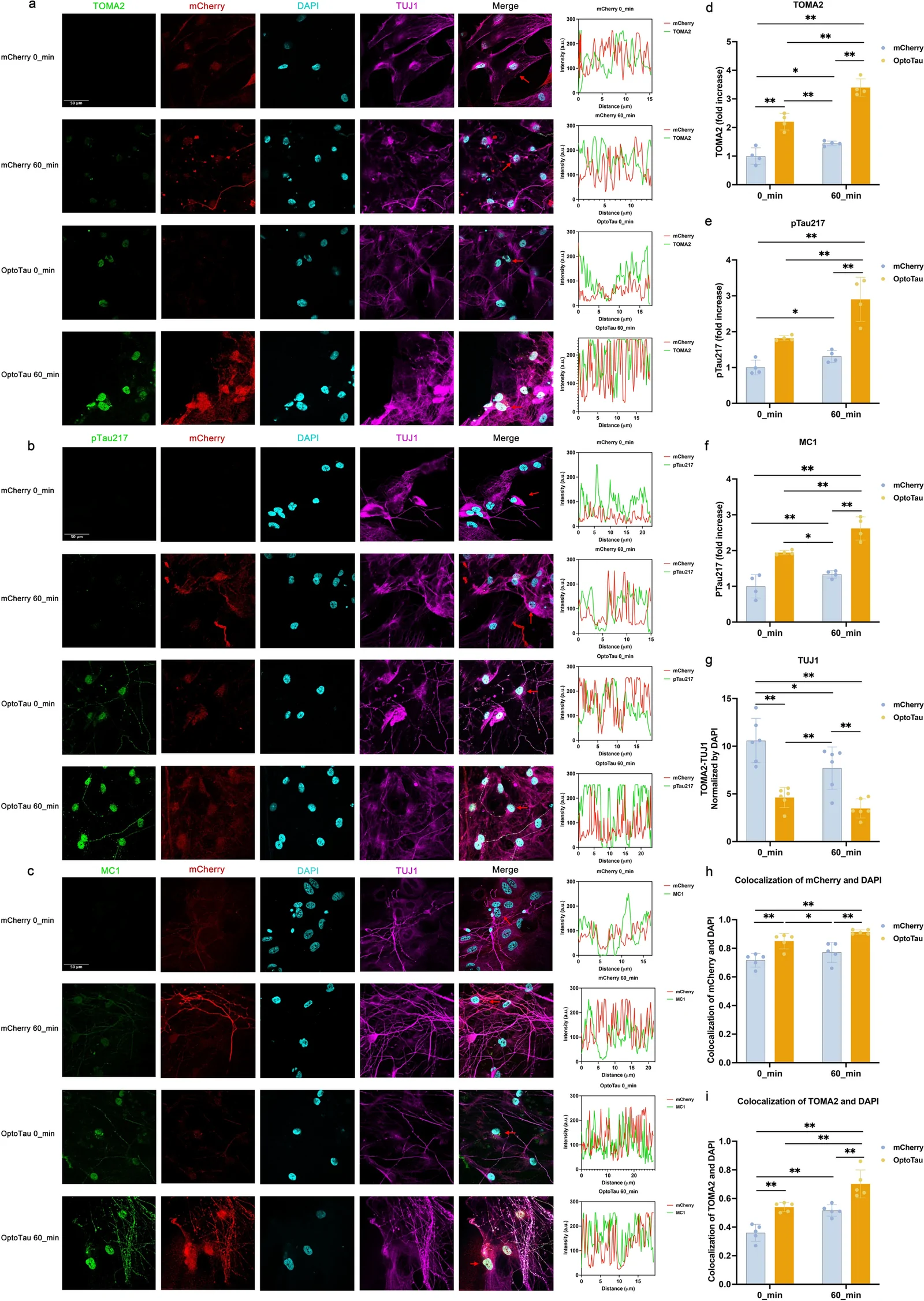
OptoTau oligomerization drives sustained accumulation of pathological tau bound to the nuclear membrane in iPSC-derived neurons. **a** Representative confocal images for colocalization of mCherry with TOMA2 in iPSC induced neurons following 0 or 60 min 488λ blue light activation (Cyan: DAPI; Red: mCherry; Green: oligomeric tau antibody TOMA2; Magenta: beta tubulin neuronal marker TUJ1). The scale bar = 50 µm. Representative histograms show fluorescence intensity of mCherry and tau marker colocalization in a single cell. **b** Representative confocal images for co-localization of mCherry with pTau217 in iPSC induced neuron. Representative histograms using phosphorylated tau marker pTau217. (Cyan: DAPI; Red: mCherry; Green: pTau217; Magenta: TUJI). **c** Representative confocal images for co-localization of mCherry with MC1 in iPSC induced neurons. (Cyan: DAPI; Red: mCherry; Green: MC1; Magenta: TUJ1). Representative histograms using the overlay peak of misfolded tau marker MC1 with mCherry. **d** Quantification for the immunofluorescence intensity fold increase of TOMA2 oligomeric tau in representative ROIs. **e** Quantification for the immunofluorescence intensity fold increase of hyper-phosphorylated tau by PTau217 antibody labeling in representative ROIs. **f** Quantification for the immunofluorescence intensity fold increase of misfolded tau by MC1 antibody labeling in representative ROIs. *N* = 4. **g** Quantification for the TUJ1 fluorescence intensity normalized by DAPI fluorescence intensity in (**a**). **h** Quantification of mCherry-DAPI colocalization in (**a**). Bar graph shows the percentage of DAPI-positive nuclei exhibiting mCherry signal. **i** Quantification of TOMA2-DAPI colocalization in (**a**). Bar graph shows the percentage of DAPI-positive nuclei exhibiting TOMA2 signal.* N* = 5. Data shown as Mean ± SEM. The two-way ANOVA with Tukey’s multiple comparisons test was used for all experiments, *p < 0.05, **p < 0.01

Our previous work has established proper functionality of OptoTau oligomerization in mouse primary cortical neurons [34], but the use of human iPSC-derived neurons offers a physiologically relevant platform for studying human AD [31, 41]. iPSC-derived neurons have the advantage of developing human neuronal features over time, including synapse formation, cell morphology, and response to neurodegenerative stressors, such as tau accumulation [31, 41]. To assess OptoTau efficacy in studying AD tau pathology in vitro, we exposed transduced neurons expressing OptoTau or mCherry to 0 or 60 min of 488λ blue light and probed for different pathological tau markers in fixed cells. Additionally, all cells were co-labeled by immuno-fluorescence of dsDNA using DAPI, mCherry to confirm vector expression, and TUJ1 to evaluate neuronal damage (Fig. 5a, b, and c).

To assess overall tau accumulation, Tau5 antibody was used for the detection of global tau [25]. Tau5 fluorescence staining confirmed strong total tau expression in OptoTau neurons and increased aggregation in OptoTau neurons with 60 min of activation and significant single cell colocalization when compared with mCherry control neurons (Supplemental Fig. S3). This confirms total tau enhancement under oTau transduction and indicates reliable expression of both constructs. Next, we assessed oligomeric tau using the TOMA2 antibody specifically targeted for neurotoxic oTau aggregates in AD [37]. As expected, TOMA2 intensity appeared highest in light activated OptoTau neurons and showed moderate histogram colocalization to the nucleus labeled by DAPI (Fig. 5a). Fluorescence quantification revealed greatest fold increases in TOMA2 among exposed OptoTau (Fig. 5d), confirming tau oligomer formation.

Further probing with pTau 217, a marker for tau phosphorylation at threonine 217, which correlates closely with AD progression [5, 32], showed close fluorescence peak overlap in OptoTau neurons following activation (Fig. 5b). The pTau 217 fluorescence also showed significant elevation in activated oTau neurons (Fig. 5e). This, in combination with its high colocalization with oTau granules makes it a suitable biomarker for tracking accumulation. Finally, misfolded tau species were assessed using the highly specific MC1 antibody that recognizes a pathological tau conformation only seen in AD afflicted neurons [36]. Once again, highest colocalization histograms with nucleus and fluorescence fold increases presented in OptoTau neurons after 60 min of blue light exposure (Fig. 5c, and f). Furthermore, in healthy brain tissue, TUJ1 (βIII-tubulin) expression reflects neuronal number and the integrity of neural microtubule structure. Therefore, it is often used as a marker for assessing neuronal damage, neurogenesis, or axonal integrity. We observed a significant decrease in TUJ1, a neurite marker, in neurons treated with OptoTau for either 0 or 60 min (Fig. 5g, and Supplemental Fig. S4). Colocalization analysis between mCherry and DAPI, together with Tau immunostaining and DAPI, demonstrated a marked increase in nuclear colocalization following 60 min of light exposure. Quantitative analysis showed that the proportion of nuclear regions positive for mCherry or Tau fluorescence was significantly higher compared with the control group. These results indicate that prolonged light exposure promotes the translocation or retention of Tau within the nucleus, implying a potential role of Tau in nuclear-associated processes under these conditions (Fig. 5h, i, and Supplemental Fig. S4).

Across all markers, we observed increased colocalization between OptoTau and pathological tau signals, along with significantly enhanced tau aggregation in light-induced cells. These findings demonstrate that OptoTau activation could initiate the formation of disease-relevant tau oligomers, phosphorylation, and conformational changes, effectively recapitulating key features of AD tauopathy in iPSC-derived neurons. The sustained accumulation of pathological tau at the nuclear membrane was observed not only with the mCherry-tagged chimeric tau (Fig. 4), but also was confirmed by additional pathological tau markers.

### Pathological tau accumulates at the invagination zone and binds to the nuclear lamina layer

**Fig. 6:**
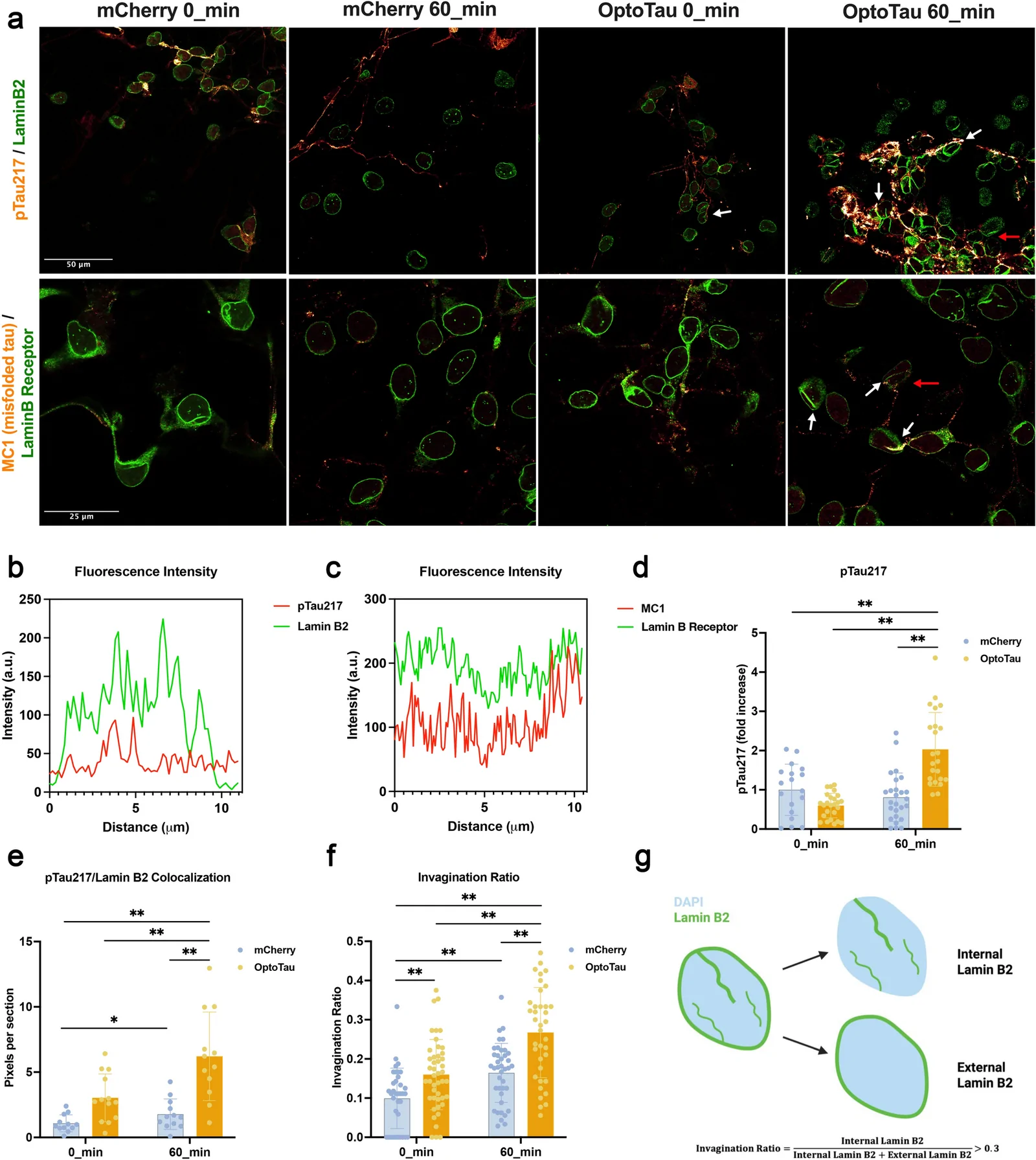
Pathological tau accumulates at the invagination zone and binds to the nuclear lamina layer. **a** Representative confocal images for colocalization of pathological tau and lamina proteins. The neurons induced by iPSC were illuminated by 488λ blue light exposed for 0 or 60 min. After 60 min of blue light activation, cells in the OptoTau condition showed significant nuclear lamin disruption, along with elevated levels of phosphorylated Tau. The pTau217 is the Tau protein phosphorylated at amino acid 217, and MC1 represents misfolding Tau. The scale bar = 50 or 25 µm as shown in the image. **b**, **c** Fluorescence intensity histogram of nuclear envelope invagination example indicated in the representative image by arrows. X axis is in units of microns (μm). **d** Quantitation for fluorescence intensity of pTau217. Higher fluorescence corresponds to integrated density normalized to DAPI dsDNA staining. *n* = 18-26. **e** Colocalization analysis of pixel % overlap between pTau217 and Lamin B2; *n* = 12-13. **f** Ratio of nuclei that present with invagination within each ROI containing 10-60 cells. Dataset derived from two separate rounds of fixing, staining, and imaging; *n* = 37-45. **g** Schematic illustrating criteria for invagination: nuclei with internal (overlapping with DAPI staining) Lamin B2 or Lamin B Receptor exceeding a proportion of 0.3 of total Lamin B2 or Lamin B Receptor are counted as invaginated. Invagination = (Internal Lamin B2)/(Internal + External Lamin B2) > 0.3. Data shown as Mean ± SEM. The two-way ANOVA with Tukey’s multiple comparisons test was used for all experiments, *p < 0.05, **p < 0.01

Recent studies indicate that perinuclear accumulation of pathological tau can induce physical nuclear distortions, including invaginations, nuclear pore complex impairment, and lamina dysfunction [20, 35, 53, 62]. These effects have been observed across multiple neurodegenerative tauopathy diseases, including AD and FTD [35, 53]. Similar lamina invaginations are also seen in non-tau-related conditions, such as Huntington’s disease, where polyglutamine aggregates are involved [42], suggesting a broader category of laminopathies, which are neurodegenerative disorders characterized by lamina layer dysfunction. Given the role of lamina proteins, particularly Lamin B2 and Lamin B Receptor, in directing chromatin architecture in neurons, elucidating patterns of nuclear disruption is critical in understanding molecular bases of disease progression in AD and related laminopathies [61, 63, 72]. To investigate the specificity of tau oligomer binding to Lamin B2 and Lamin B Receptor, as opposed to general protein aggregation, we used the same mCherry and OptoTau constructs as shown in live-cell time-lapse imaging of iPSC-derived neurons to examine how pathological tau progressively interacts with the nuclear lamina in iNeurons. To assess whether the nuclear deformation induced by OptoTau oligomerization is reversible, we performed a light cycling induction of oligomerization followed by immuno-fluorescence co-labeling for dsDNA by DAPI, lamina components by LB2 and LBR antibodies, pathological tau and neuronal markers, respectively (Fig. 6a). Specifically, after lentiviral transduction, iPSC-derived neurons were exposed to blue light for 60 min in a pulsed manner: 20 min of light exposure followed by 20 min without light, repeated 3 × . Cells were then fixed and stained for nuclear envelope markers. Our results showed that in the mCherry-only control condition, nuclear morphology remained unaffected under this light cycling protocol. However, in neurons expressing OptoTau, significant nuclear invagination persisted even during the recovery intervals when the light was off. This suggests that once tau oligomerization is induced, the associated nuclear deformation is not readily reversible within the time frame of our experiment.

Our results indicate commonalities between live-cell observations and continuous aggregation of tau in cell nuclear morphologies (Figs. 4, 5). In the current experiment, we used fluorescence mapping of Lamin B2 with pTau 217 and Lamin B Receptor with MC1 to evaluate the presence of lamina folds overlapping with pathological tau accumulation (Fig. 6a-c). Fluorescence intensity histograms reveal peak overlaps of pTau 217 with Lamin B2 and MC1 misfolded tau marker with Lamin B Receptor invagination (Fig. 6b, c). As expected, the activated OptoTau neurons presented with elevated levels of phosphorylated tau, aligning with established mechanisms of tau oligomer propagation by post-translational modification (Fig. 6d) [5, 32]. Colocalization analysis of pTau 217 with Lamin B2 confirmed significantly increased levels of interaction between phosphorylated tau and Lamin B2, suggesting mislocalization of tau to nuclear lamina (Fig. 6e). We also used Lamin B2 within the internal nuclei as an indicative of nuclear invagination. Nuclei with internal Lamin B2 at a ratio greater than 0.3 of total Lamin B2 at each nucleus qualified as invaginated (Fig. 6f, g) [53]. Examining ratios of nuclei presenting with invagination in each ROI, we find the highest rates of nuclear invagination in OptoTau cell culture following blue light activation (Fig. 6f). These findings demonstrate that upon oligomerization, tau aggregates localize to the nuclear membrane and exert mechanical pressure on the lamina nucleoskeleton, potentially impairing its functionality.

### Accumulation of tau and interactions with the nuclear lamina coincide with dysregulated protein synthesis in iPSC-derived neurons

**Fig. 7:**
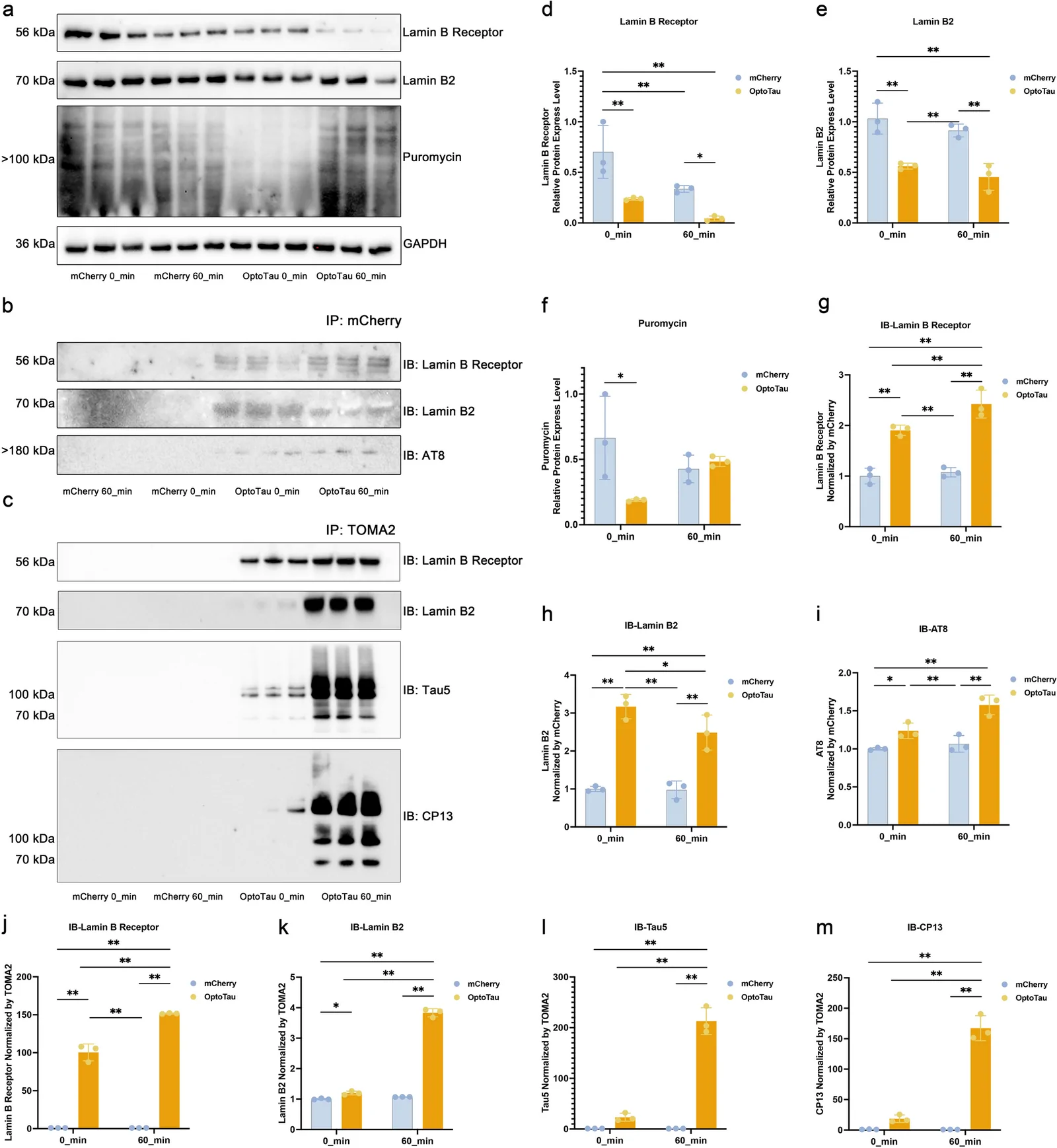
OptoTau interacts with the nuclear lamina and is associated with dysregulated protein synthesis in iPSC-derived neurons. **a** Representative Western blot analysis of nuclear membrane proteins and nascent proteins in iPSC-induced neurons. Total cell lysates were collected from iPSC-induced neurons under control and optogenetic induction. Blots were probed with antibodies against the nuclear membrane proteins Lamin B Receptor, Lamin B2, Lys9, and the nascent protein label Puromycin (via anti-Puromycin antibody). Antibody against GAPDH was used as the loading control. The images demonstrate a concomitant reduction in nuclear envelope integrity and protein synthesis. **b** Co-Immunoprecipitation of the optogenetically induced tau complex reveals direct interaction with nuclear membrane proteins. Lysates from iPSC-induced neurons expressing the optogenetic tau construct (fused to mCherry) were subjected to immunoprecipitation by ChromoTek RFP-Trap^®^ Agarose. This allows for an unbiased isolation of proteins complexed with tau. The presence of AT8, Lamin B2 and Lamin B Receptor in the immunoprecipitated complex was assessed by Western blot, indicating a direct or close-association binding between oligomeric tau and these nuclear membrane components. **c** Co-Immunoprecipitation of the optogenetically induced tau complex reveals direct interaction with nuclear membrane proteins. Lysates from iPSC-induced neurons expressing the optogenetic tau construct (fused to mCherry) were subjected to immunoprecipitation by TOMA2. The presence of Tau5, CP13, Lamin B2 and Lamin B Receptor in the immunoprecipitated complex was assessed by Western blot, indicating a direct or close-association binding between oligomeric tau and these nuclear membrane components. **d-f** Quantification of Western blot results shown in (**a**). Band intensities were quantified using ImageJ and normalized to the corresponding GAPDH loading control. Data are presented as fold-change relative to the mean of the control group (set to 1.0). **g-i** Quantification of co-IP results shown in (**b**). AT8, Lamin B2 and Lamin B Receptor enrichment in the mCherry-tau immunoprecipitate. The band intensity for each protein in the co-IP lane was quantified for pulldown efficiency. **j-m** Quantification of co-IP results shown in (**c**). Tau5, CP13, Lamin B2 and Lamin B Receptor enrichment in the TOMA2 immunoprecipitate. The band intensity for each protein in the IP lane was quantified for pulldown efficiency. Data are presented as mean ± SEM. Statistical significance was determined using two-way ANOVA followed by Tukey’s multiple comparisons test. *p < 0.05, **p < 0.01, *n* = 3

To further investigate the interaction between pathological tau and nuclear lamina, we conducted Western blot and co-immunoprecipitation (co-IP) assays using human iPSC-derived neurons expressing OptoTau. Following 60 min of blue light stimulation (three cycles of 20-min light exposure separated by 20-min intervals without light), Western blot analysis of neuronal total lysates revealed a significant increase in pathological tau species, including TOMA2, AT8 and pTau217, consistent with robust tau oligomerization and aggregation (Supplemental Fig. S5a-d). Concurrently, levels of nuclear membrane proteins Lamin B2 and Lamin B Receptor were significantly reduced under OptoTau activation conditions shown by quantitative densitometry analysis (Fig. 7a, d and e). These results further confirmed a causative tau-mediated disruption of nuclear envelope integrity.

To evaluate whether tau aggregation induces epigenomic changes, we examined the expression of H3K9me3 (Histone H3 Lysine 9 trimethylation), which is a well-established epigenetic marker of DNA damage response and heterochromatin formation, in human-induced iPSCs. Western blot analysis was performed on all experimental groups following a standardized 60-min light exposure protocol. Despite extended film exposure durations, only minimal immunoreactive bands were observed across all samples (Fig. 7a). The consistently low-signal intensity of H3K9me3 fell below the quantitative detection limit, precluding statistical comparison between groups. Critically, the absence of upregulated H3K9me3 expression in light-exposed cells suggests that neither the 60-min photostimulation regimen nor oligomeric tau aggregation elicits detectable DNA damage or sustained epigenetic stress in iPSCs under these experimental conditions. To further investigate whether oligomeric tau-lamina interactions impact transcriptional dysregulation [17, 35], as an early driver of pathology, we examined protein translation with puromycin in the iPSC-derived neurons with surface sensing of translation (SUnSET) methodology [56]. The results suggest that tau oligomerization may confer distinct functional properties in regulating protein synthesis following nuclear invagination, compared with monomeric tau (Fig. 7a, f).

To add evidence for the direct binding between tau and nuclear lamina components, we performed co-immunoprecipitation using an unbiased anti-mCherry antibody targeting the OptoTau-mCherry construct. As shown in Fig. 7b, Lamin B2 and Lamin B Receptor were co-precipitated with mCherry, supporting a direct or close association between tau oligomers and nuclear membrane proteins. Densitometric quantification of co-precipitated proteins is shown in Fig. 7g-i, further validating tau’s engagement with nuclear lamina components upon oligomerization. To more specifically assess the interaction of Lamin B2 and LBR with oligomeric tau species, we performed additional co-immunoprecipitation experiments using the oligomer-specific tau antibody TOMA2 (Fig. 7c). This approach selectively enriches oligomeric tau while minimizing the contribution of monomeric OptoTau present under non-light-induced conditions. We then probed for Lamin B2 and LBR to evaluate their association with oligomeric tau. The results demonstrated a specific and robust interaction between Lamin B2 and oligomeric tau under light-induced conditions. LBR similarly showed a stronger association in the light-induced condition compared with the no-light OptoTau condition (Fig. 7c and j, k). We also confirmed enrichment and pulldown of oligomeric tau complexes by immunoblotting with the total tau antibody Tau5 and the phospho-tau antibody CP13 (Fig. 7c and l, m). In the OptoTau with light condition, prominent high-molecular weight tau species (> 150 kDa) were detected, which were absent in the mCherry::Cry2Olig control condition. Tau5-immunoreactive bands spanned approximately 70-250 kDa, with enrichment around ~ 130 kDa, whereas CP13-positive phospho-tau species were predominantly enriched above 150 kDa, representing high molecular weight oligomeric forms. Additionally, to determine how early Cry2Olig-mediated opto induction of chimeric tau oligomerization leads to tau phosphorylation while excluding potential cellular stress effects from the mCherry::Cry2Olig control, we performed additional experiments using a 20-min illumination paradigm. A 20-min light exposure did not induce pTau217 or AT8-positive tau phosphorylation under mCherry::Cry2Olig control conditions. In contrast, OptoTau-expressing cells showed a clear increase in tau phosphorylation following light induction (Figure S5e-g). These results further confirm that illumination of mCherry::Cry2Olig alone does not induce detectable increases in tau phosphorylation and that a 20-min light exposure is sufficient to trigger OptoTau oligomerization and associated phosphorylation.

## Discussion

While the presence of tau aggregation has been extensively identified in multiple neurodegenerative diseases [4, 19, 45, 57, 73], the precise mechanisms by which tau aggregates induce cellular dysfunction and toxicity remains enigma. Furthermore, the role of nuclear destabilization, a process observed in AD and related tauopathies, in initiating and driving disease progression, or occurring as a consequence of neurodegeneration, is a paradox [53]. The findings presented in this study fill this knowledge gap by providing comprehensive evidence that tau oligomer species initiate and drive nuclear membrane disruption as an early and contributing mechanism in AD pathogenesis. Utilizing a robust, multimodal approach with an optogenetic construct in human iPSC-induced neurons, transgenic mouse models, and human AD post-mortem brain tissue, we find that tau oligomers localize to and interact with nuclear lamina proteins Lamin B2 and Lamin B Receptor, thereby leading to invagination, compromised nuclear integrity, and chromatin decompaction. These findings align with recent studies implicating tau in toxic effects on the nucleus [20, 34, 53] and offer new insight into early pathological mechanisms of aggregate-lamina disruption across multiple neurodegenerative diseases [35].

Our most clinically relevant findings are from examination of human post-mortem AD brain tissue. The findings from human brain tissue align with experimental models. Immunofluorescence co-labeling finds concordance between emergence of MC1-positive misfolded tau and loss of Lamin B Receptor nuclear integrity beginning in Braak stage III, well before large-scale tau tangle deposition and neurodegeneration. The temporal alignment emphasizes that nuclear membrane disruption is a precursor to late-stage degeneration and is critical in identifying the nuclear lamina as a potential biomarker and therapeutic target in preclinical AD [23]. Lamin B Receptor loss progressed from controls to severe AD cases, matching tauopathy severity in PS19 mice and iPSC-neuron systems. The cross-model consistency confirms nuclear membrane pathology as an early AD trigger. It likely initiates transcriptional cascades that worsen tau toxicity [18]. Therapies aimed at lamina stabilization could mitigate chromatin and transcriptional dysregulation before degeneration takes root. Similar approaches hold precedent in progeria research where lamina-stabilizing compounds have shown promise in mitigating nuclear pathology [7], but similar strategies adapted to AD tau-induced nuclear aging warrant further research and exploration [38, 44]. Importantly, although age-related nuclear alterations have been reported in multiple tissues, our additional analyses indicate that LBR reduction in human AD cortex is not merely a function of chronological aging. Neither continuous age analysis nor age stratification revealed a significant association between age and Lamin B Receptor levels. In contrast, diagnostic group was a significant determinant of LBR reduction. These findings suggest that nuclear lamina disruption reflects disease-associated nuclear pathology rather than general age-related decline. This distinction is particularly important given the advanced age of AD patients and underscores the tau pathology specificity of nuclear lamina alterations in AD.

Findings in PS19 tau transgenic mice confirmed an association between tau pathology and nuclear lamina invagination. Nuclear invagination and pTau217 intensity were significantly more prevalent in aged PS19 mice, with trends in 5-month PS19 resembling 9-month WT, this overlap indicates that these nuclear alterations precede overt neurodegeneration and may represent an early manifestation of neuronal stress associated with aging or/and tau pathology. Comparable nuclear envelope abnormalities have been reported in physiological neuronal aging as well as in laminopathies, such as Hutchinson-Gilford Progeria Syndrome, suggesting that lamina perturbation likely represents a shared cellular response rather than a disease-specific hallmark [26, 43, 44]. The strong positive correlation between nuclear tau localization and lamina disruption observed selectively in PS19 mice supports the view that tau pathology modulates the downstream consequences of nuclear stress, thereby increasing neuronal susceptibility to neurodegeneration. Consistent with this interpretation, chromatin reorganization emerged as the most prominent downstream alteration in hippocampal neurons of PS19 mice. The nuclear lamina is critical in chromatin structure and stability via lamina-associated domains (LADs) which tether heterochromatin to the nuclear periphery [15, 65]. Given the central role of the nuclear lamina in maintaining chromatin organization through lamina-associated domains that anchor heterochromatin to the nuclear periphery, lamina disruption in the presence of pathological tau may contribute to altered chromatin homeostasis without being sufficient to trigger neurodegeneration by itself. Our results indicate neurons presenting with elevated tau-induced lamina intrusion show increased chromatin coverage and decreased heterochromatin density, particularly in the LAD-rich NMZ. The observed chromatin decompaction in PS19 mice, a nuclear stress response, precedes cell death and, therefore, is likely not secondary to neurodegeneration, again emphasizing that nuclear and chromatin disruption are early and causative to neurodegeneration rather than following it [21, 59]. We propose a tauopathy cascade: (1) Tau oligomers induce nuclear invaginations by disrupting the lamina. (2) Membrane deformations destabilize lamina-associated chromatin domains. (3) Chromatin decompaction drives transcriptional dysfunction, accelerating neurodegeneration. Tau pathology mimics accelerated nuclear aging [48, 49]. Physiological aging processes that take decades are compressed into months. This highlights nuclear integrity’s critical role in neuronal survival. Besides, it should be noted that age and amyloid burden may influence nuclear lamina integrity. Although our analyses were not stratified by these variables, we acknowledge them as potential confounders and interpret the observed nuclear lamina changes with caution.

Our optogenetic induction of 4R1N Tau oligomerization in human iPSC-derived neurons replicated key AD features, including pathological tau aggregation, phosphorylation at Thr217, and disease-like conformational changes labeled by MC1. This highly controllable platform enables analysis of tau’s spatial temporal trafficking and interactions by live-cell imaging. The live-cell time-lapse imaging showed that tau oligomerization, an early process in tauopathies [5, 22, 32, 46], initiated rapid and significant nuclear membrane deformation. The decreases in nuclear circularity observed in 30 min of activation were specific to OptoTau-expressing neurons, confirming resulting structural abnormalities are not artifacts of Cry2Olig activation or protein overexpression but are tau-aggregation-dependent phenomena. Rapid nuclear targeting aligns with recent work demonstrating tau oligomers possess high conformational mobility and a tendency to interact with off-target proteins and hydrophobic domains such as those seen in the lipid-rich double layer nuclear membranes [11, 22, 32, 46]. Consistent with our findings, previous studies have demonstrated that TUJ1, as the βIII-tubulin, is significantly reduced in AD [9, 54]. The decrease of fluorescence intensity in this neuron-specific microtubule marker reflects neuronal loss and cytoskeletal disruption, both of which are hallmarks of AD pathology. Aβ accumulation and hyperphosphorylated tau can destabilize microtubules, leading to the degradation or mislocalization of βIII-tubulin. Moreover, impaired neurogenesis in the hippocampus further contributes to reduced TUJ1 expression. Together, these findings suggest that OptoTau downregulated TUJ1 expression downregulation, indicating neurodegenerative progression in AD. Further colocalization analyses of mCherry and DAPI, as well as Tau-specific immunostaining with DAPI, revealed a pronounced increase in nuclear colocalization after 60 min of light exposure. The enhanced overlap of mCherry and Tau signals with nuclear DAPI staining suggests that light stimulation facilitates the accumulation of Tau within the nucleus. Given that nuclear Tau has been implicated in chromatin organization, DNA protection, and transcriptional regulation, our findings raise the possibility that light-induced nuclear localization of Tau may contribute to alterations in nuclear homeostasis or gene expression in this model.

To assess whether the nuclear deformation induced by OptoTau oligomerization is reversible, we performed a light cycling experiment during the immunostaining protocol. Our results showed that in the mCherry-only control condition, nuclear morphology remained unaffected under this light cycling protocol. However, in neurons expressing OptoTau, significant nuclear invagination persisted even during the recovery intervals when the light was off. This suggests that once tau oligomerisation is induced, the associated nuclear deformation is not readily reversible within the time frame of our experiment. Furthermore, our immunoprecipitation and Western blot experiments confirmed tau’s physical interaction with nuclear lamina proteins following both a single 20-min light exposure and three repeated 20-min illumination cycles (Fig. 7 and Supplemental Fig. S5e). We note that 20 min of illumination minimized baseline effects in the mCherry-Cry2Olig control condition. However, repeated mCherry-Cry2Olig aggregation induced a slight increase in tau phosphorylation, likely due to the extended duration of inducible protein aggregation in neurons. Importantly, repeated OptoTau aggregation resulted in pronounced nuclear membrane damage compared with the mCherry-Cry2Olig control condition, demonstrating a robust and tau-dependent effect. These structural defects likely drive neurodegeneration through four pathways: the activation of nuclear stress responses, the disruption of chromatin organization, the altered gene expression, and the impaired nucleocytoplasmic transport [17, 35]. Together, these combined evidence emphasize that tau’s localization to nuclear boundaries and interaction with lamina components are not incidental but mechanistically integral to its neurotoxicity. Most importantly, the early emergence of tau oligomers and nuclear localization are consistent with theories affirming oTau as the primary toxic drivers in AD, preceding and potentiating neurofibrillary tangles [58, 67]. Our study supports the ongoing paradigm shift in AD research away from late stage accumulated fibrillary tau and amyloid beta plaques to early-stage oligomer mediated disruption [26, 38]. These insights open new avenues for therapeutic development in tauopathies [3, 38, 41]. In the puromycin assay, mCherry-expressing cells showed relatively high signal at baseline (0 min) that slightly decreased after blue light exposure, whereas Opto tau-expressing cells exhibited low baseline signal that modestly increased at 60 min, approaching the levels seen in mCherry cells under the same conditions. These observations indicate that alterations in global protein synthesis depend on both the expressed construct and the oligomerization state of tau. Notably, we observed dynamic changes in protein translation rates during the transition from monomeric to oligomeric tau, suggesting that tau oligomerization might confer functional properties distinct from those of monomeric tau.

The mCherry pull-down and TOMA2 pull-down results in Fig. 7 indicate that tau can interact with Lamin B2 and LBR in a non-oligomerized state, while oligomerization appears to enhance or stabilize these interactions. In light of these considerations, the conclusions drawn from Fig. 7 are limited to demonstrating context-dependent tau-lamina interactions and associated changes in protein phosphorylation and translation, without implying that oligomerization is necessary for binding or for the observed translational effects. Our findings indicate that short-term light exposure (20 min) minimizes off-target toxicity associated with the mCherry-Cry2Olig illumination protocol. Specifically, mCherry-Cry2Olig alone did not exhibit detectable increases in pTau217 or AT8 under these conditions, whereas mCherry-tagged tau oligomers displayed marked elevation of these phosphorylation markers. The observed tau phosphorylation is driven by tau oligomerization rather than light-induced non-specific effects. Thus, the 20-min or recycles of 20-min protocol provide a robust approach for inducing tau oligomers while limiting potential phototoxicity.

**Fig. 8:**
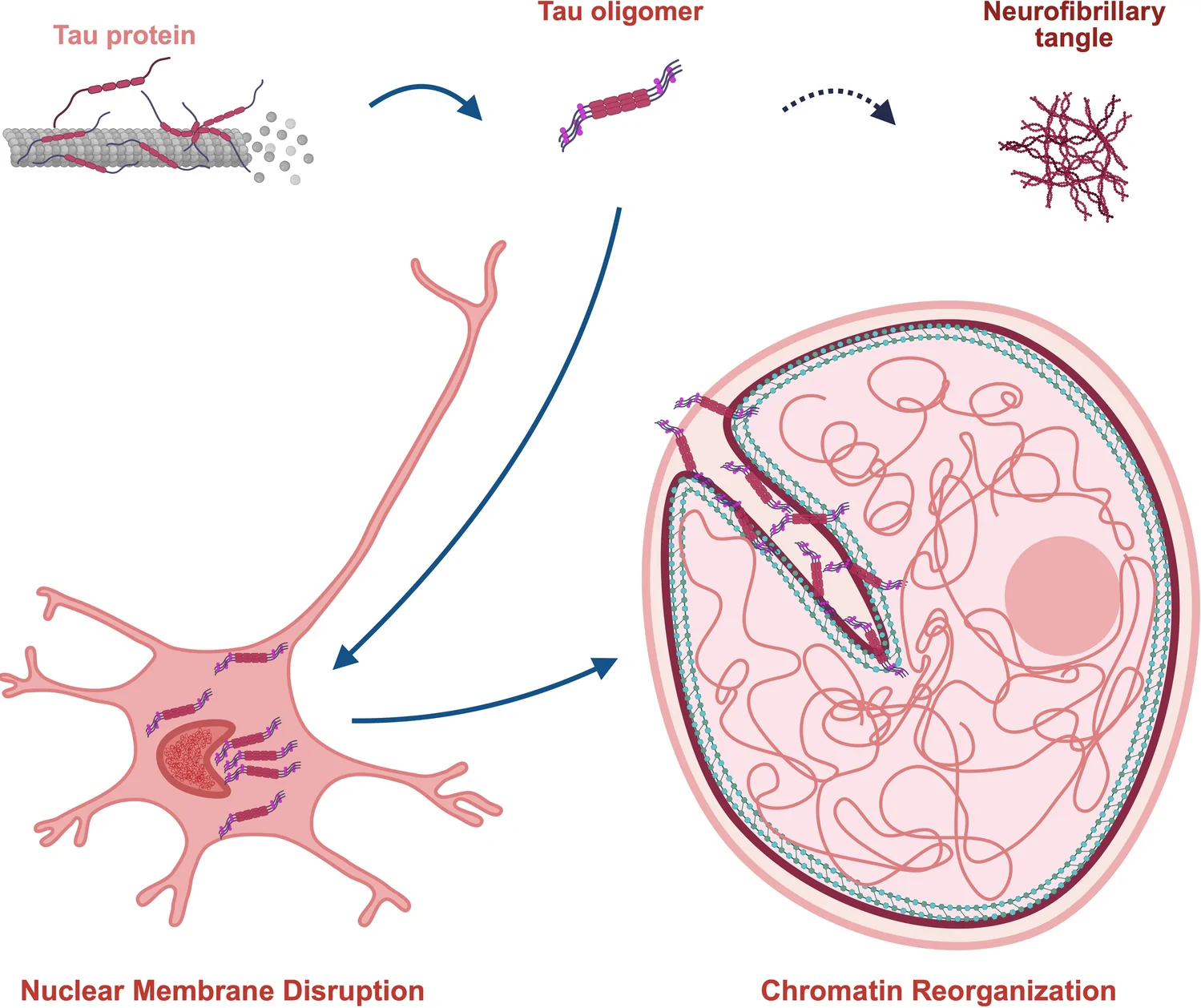
The Crosstalk between Tau Aggregation and Nuclear Membrane Disruption in Alzheimer’s disease. Early pathological tau protein oligomerization promotes nuclear lamina invagination, leading to chromatin remodeling and epigenomic alterations. This feedback loop may exacerbate neuronal stress and promote the progression of neurodegenerative diseases

Collectively, our findings reframe the understanding of tau-mediated neurodegeneration by identifying the nuclear membrane as a substantial target of early tau toxicity. Our nuclear-centric model, supported by findings in human, mouse, and cell culture systems integrates structural biology, chromatin dynamics, and previous neurodegeneration understanding into a unified pathogenic cascade (Fig. 8). Under this model, AD and potentially other neurodegenerative conditions are conceptualized as “nuclear membrane diseases,” involving aggregate-induced mechanical destabilization of genome integrity, thereby precipitating neuronal decline. By detailing these findings, this work served as a compelling rationale for nuclear targeted therapeutic strategies aimed at restoring lamina function and genome integrity. Although our study did not directly assess gene expression changes, previous work suggests that tau-mediated nuclear stress could potentially influence transcriptional regulation. Here, we consider gene expression dysregulation as a speculative pathway that may contribute to neurodegeneration, but further studies are needed to test this hypothesis [70]. Our findings corroborate and extend existing literature on mechanical perspectives of AD research and redirect attention toward previously underappreciated nuclear targets.

In conclusion, our study demonstrates that tau oligomerization directly induces nuclear membrane invagination, rupture, and disruption of the nuclear lamina, identifying a potential central mechanism underlying neuronal degeneration in tauopathies such as AD. Tau-induced nuclear abnormalities also lead to alterations in chromatin spatial organization, potentially contributing to dysregulation of gene expression and protein translation. In brain tissue from AD patients, nuclear lamina disruption is significantly correlated with pathological tau deposition and precedes symptomatic stages, suggesting its potential as a biomarker for early diagnosis or disease staging. Our optogenetic induction system enables precise spatial and temporal control of tau oligomerization, establishing a technical foundation for high-throughput drug screening targeting this process. Therapeutic strategies aimed at preventing tau oligomerization or blocking its interaction with nuclear lamina proteins may offer novel avenues to slow the progression of tauopathies, including AD.

## Supplementary Information

Below is the link to the electronic supplementary material.

Supplementary file1 (DOCX 10637 KB)

Supplementary file2 (MOV 2382 KB)

Supplementary file3 (MOV 2521 KB)

## Data Availability

All data reported in this paper will be shared by the lead contact upon request. The raw IF, EM, DAB and WB images will be made publicly available at Mendeley Data (Reserved https://doi.org/10.17632/ycb9n7gv8f.1).
