# Correction: Tau oligomerization induces nuclear lamina invagination and chromatin remodeling in Alzheimer’s disease

**DOI:** 10.1007/s00401-026-03092-5

**Published:** 2026-10-21

**Authors:** Shuo Yuan, Nicholas Essepian, Rebecca Roberts, Eliana Sherman, Weronika M. Gniadzik, Qingbo Wang, Alev Erisir, Lulu Jiang

**Affiliations:** 1 Department of Neuroscience, University of Virginia School of Medicine, Charlottesville, VA 22908 USA; 2 Center for Brain Immunology and Glia (BIG), University of Virginia School of Medicine, Charlottesville, VA 22908 USA; 3 Department of Psychology, University of Virginia, Charlottesville, VA 22904 USA; 4 Department of Neurology, University of Virginia School of Medicine, Charlottesville, VA 22908 USA

**Correction: Acta Neuropathologica (2026) 151:43** **https://doi.org/10.1007/s00401-026-03018-1**

In the original version of this article, the author name “Weronika M. Gniadzik” was missed in the author group. Now, it has been included in the author group.

The original article has been corrected.

