# Supplementary material for: Tau oligomerization induces nuclear lamina invagination and chromatin remodeling in Alzheimer’s disease
Source: Acta Neuropathol. 2026 Apr 22;151(1):43. doi: 10.1007/s00401-026-03018-1 (PMC13102949; doi:10.1007/s00401-026-03018-1)
Supplement: Supplementary file 1 — Supplementary file1 (DOCX 10637 KB) [file 401_2026_3018_MOESM1_ESM.docx]

**Tau Oligomerization Induces Nuclear Lamina Invagination and Chromatin Remodeling in Alzheimer’s Disease**

**Shuo Yuan ^1, 2#^, Nicholas Essepian ^1#^, Rebecca Roberts ^3^, Eliana Sherman ^1, 2^, Qingbo Wang ^1, 2^, Alev Erisir ^3^, Lulu Jiang ^1, 2, 4*^**

^1^ Department of Neuroscience, ^2^ Center for Brain Immunology and Glia (BIG), University of Virginia School of Medicine, VA, USA 22908

^3^ Department of Psychology, University of Virginia, Charlottesville, VA, USA 22904

^4^Neuroscience Graduate Program, University of Virginia, Charlottesville, Virginia, USA

* Correspondence should be addressed to: jiang.lulu@virginia.edu

**^#^ These authors contributed equally.**

**Supplementary Materials**

**Supplementary Material-1:**

**Supplemental video-1:** Live cell imaging of 30 minutes of blue light activation shows that mCherry particles move about randomly within the cell.

**Supplementary Material-2**

**Supplemental video-2:** Live cell imaging of 30 minutes of blue light activation shows that OptoTau granules quickly aggregated toward the nuclear envelope, leading to nuclear deformation.

**Supplementary Material-3**


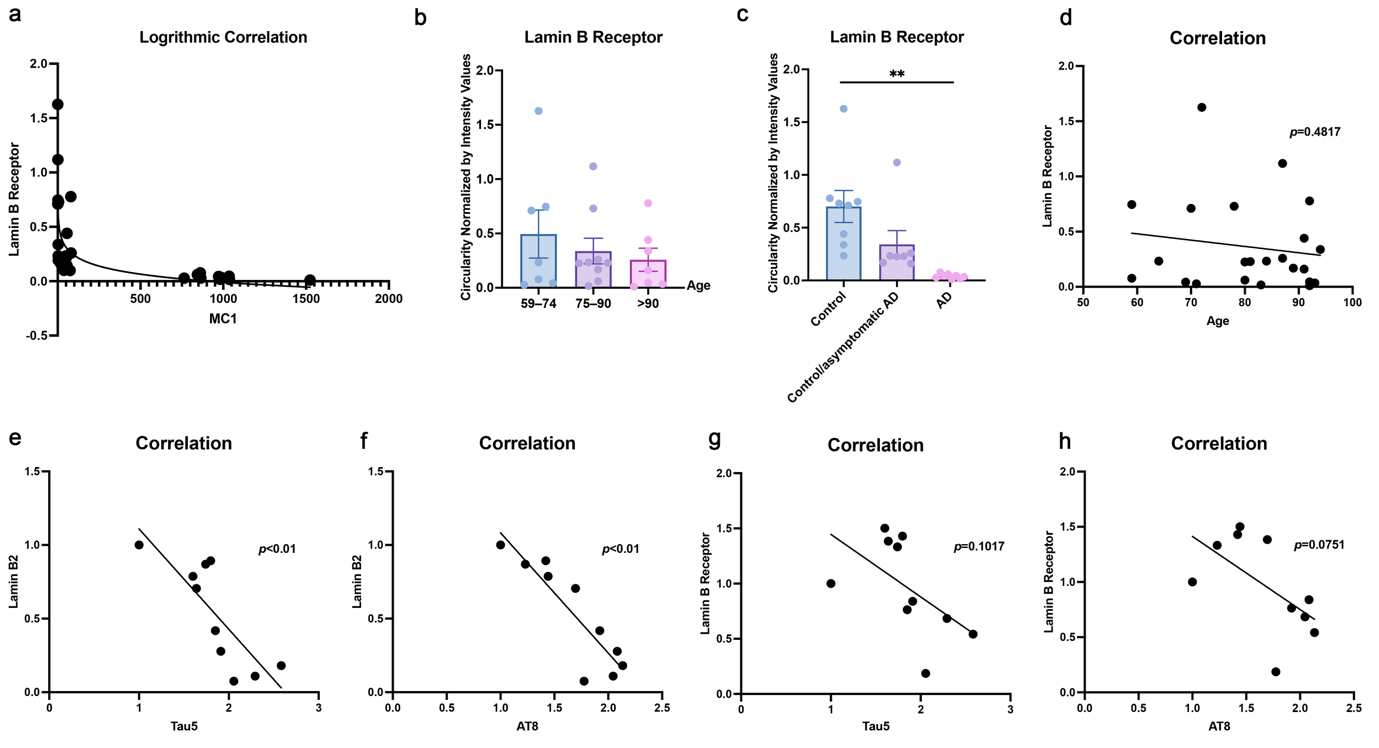


**Supplementary Figure 1. Additional statistical analyses of Lamin B and Lamin B Receptor levels in human brain samples.**

**a)** Logarithmic regression analysis corresponding to ***Figure 1e***. Logarithmic fitting was performed to evaluate the potential non-linear relationship between MC1 and Lamin B Receptor.

**b)** Quantification of Lamin B Receptor levels stratified into three age groups (59-74, 75-90, and >90 years old). One-way ANOVA showed no significant differences among groups (*p* = 0.5710, *n*≥7).

**c)** Comparison of LBR levels among control, control/asymptomatic AD, and AD cases. One-way ANOVA revealed a significant reduction in LBR levels in AD compared with controls (*p* = 0.0019, *n*≥7).

**d)** Scatter plot showing LBR levels plotted against age for all cases. Linear regression analysis revealed no significant correlation between LBR levels and age (*p* = 0.5710, *n*≥7).

**e-f)** Correlation between Lamin B2 levels and Tau5 or AT8. Lamin B2 levels were significantly negatively correlated with both AT8 and Tau5.

**g-h)** Correlation between Lamin B receptor levels and Tau5 or AT8. Although no statistically significant correlations were observed, Lamin B receptor levels exhibited a decreasing trend with increasing tau levels.

**Supplementary Material-4**


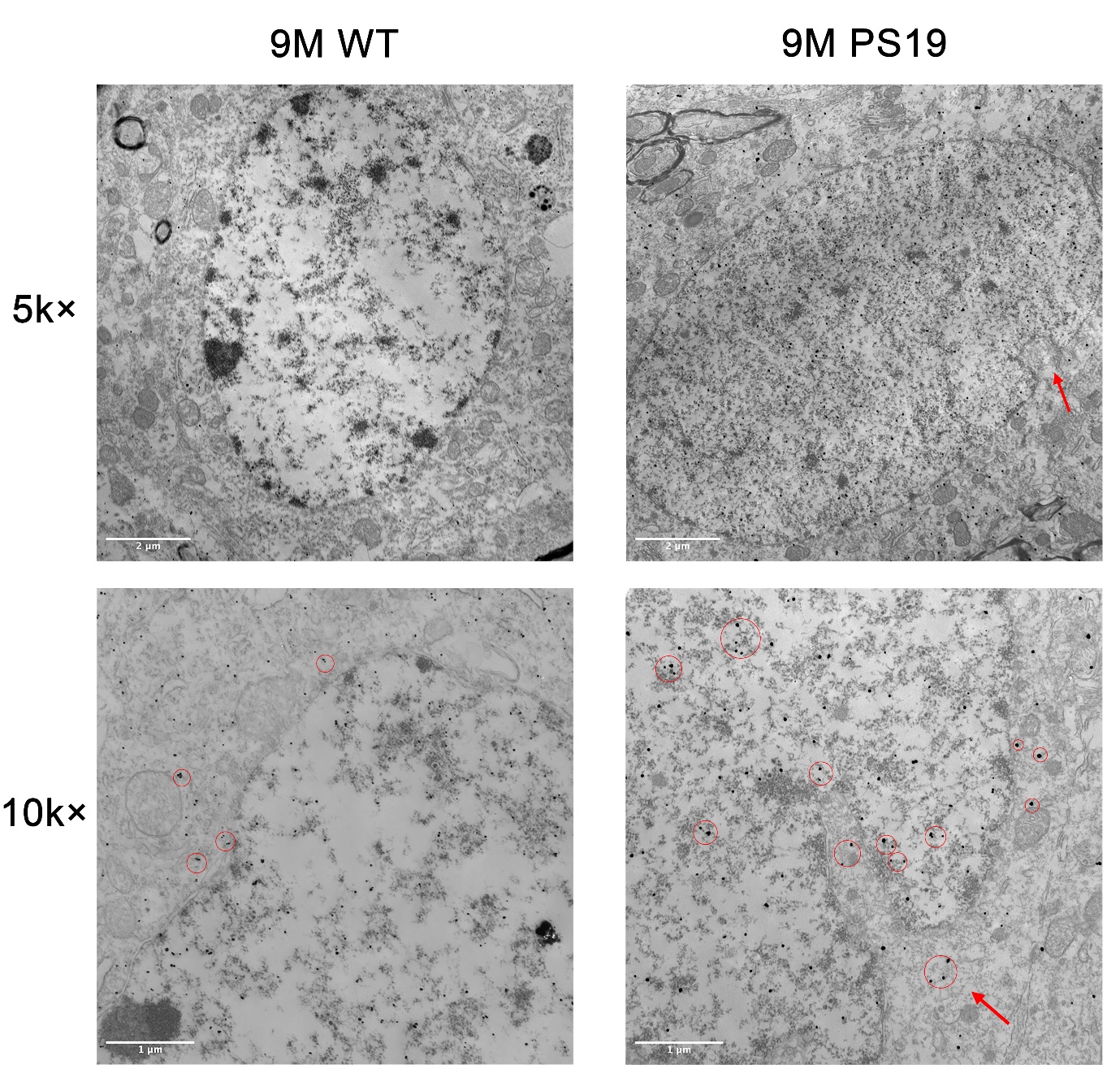


**Supplemental Fig. 2. Representative micrographs showing MC1 immunostaining in WT and PS19 mice brain sections processed with gold-silver staining to visualize pathological Tau conformers.** Images acquired at 5k× and 10k× magnifications highlight the distribution and morphology of silver-positive neuronal structures. Arrows indicate positive gold labeling with silver-enhanced immuno-EM signal corresponding to MC-1-positive tau aggregates.

**Supplementary Material-5**


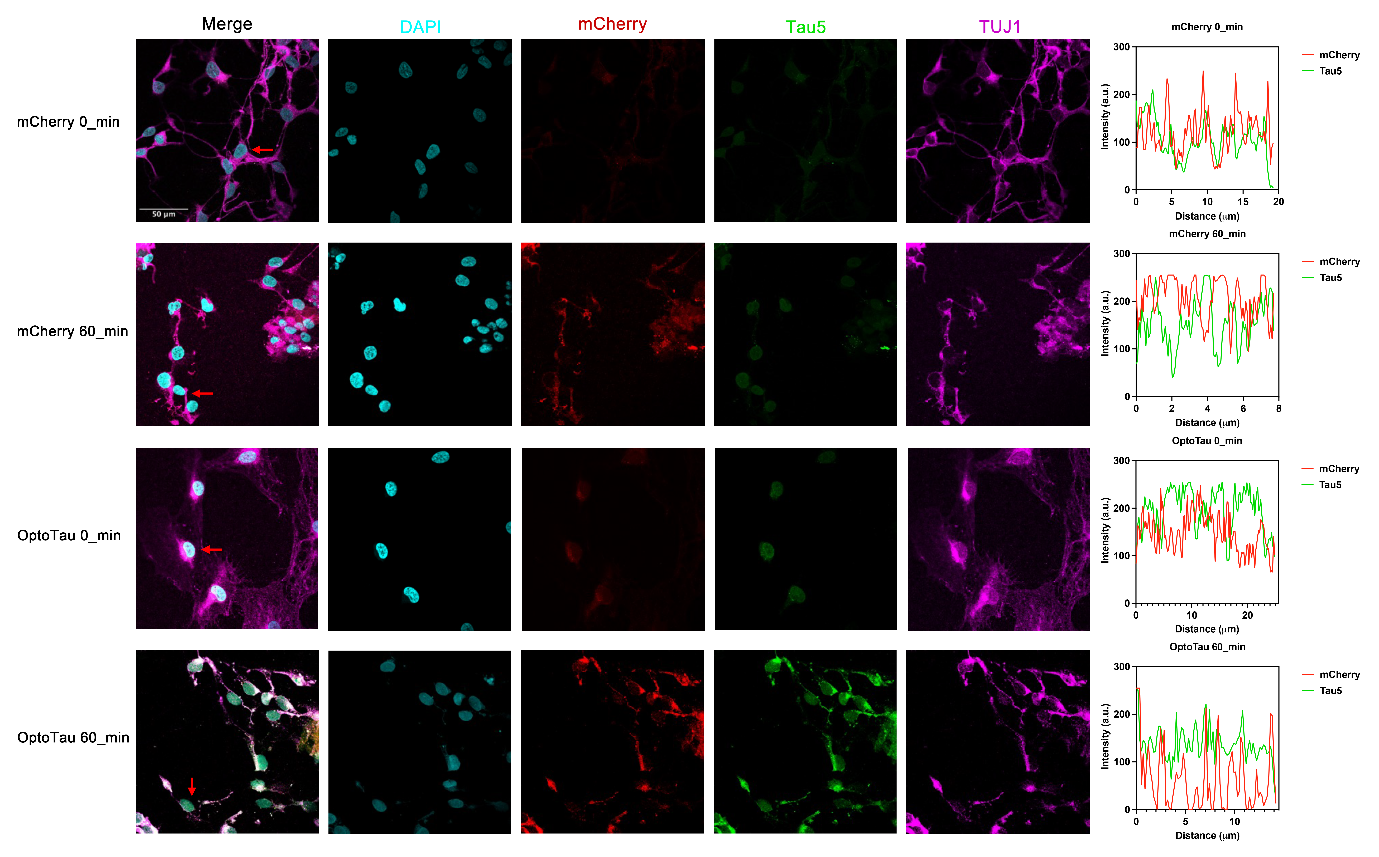


**Supplemental Fig. 3. OptoTau expressing neurons show pathological tau accumulation following prolonged 488λ blue light activation examined by Tau 5 antibody**. Representative confocal images for co-localization of mCherry with Tau5 positive total tau level in iPSC induced Neuron. (63× magnifications, Cyan: DAPI; Red: mCherry; Green: Tau5; Magenta: TUJ1). The scale bar=50 µm.


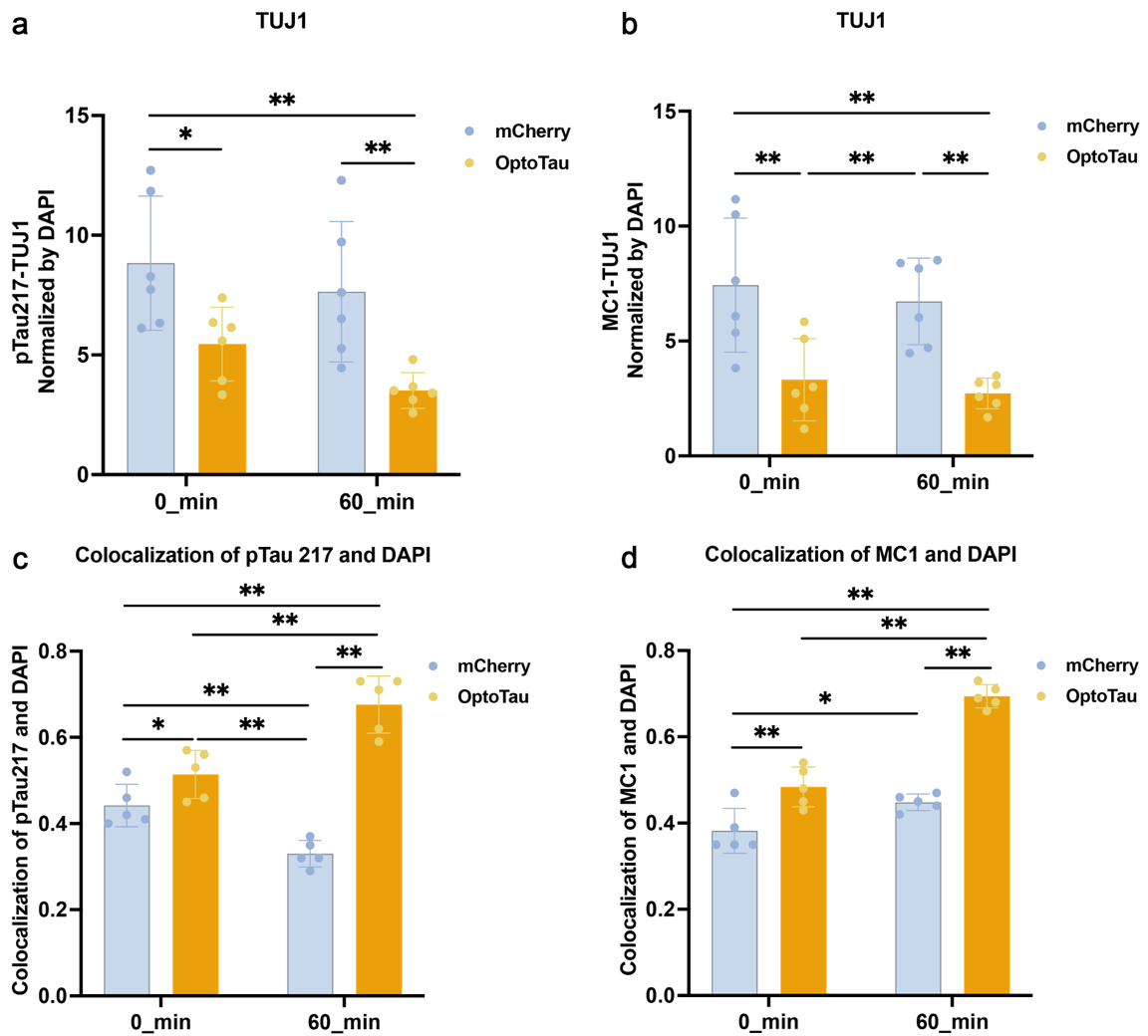
**Supplementary Material-6**

**Supplemental Fig. 4. OptoTau oligomerization drives sustained accumulation of pathological tau bound to the nuclear membrane in iPSC-derived neurons.**

**a)** Quantification for the TUJ1 fluorescence intensity normalized by DAPI fluorescence intensity in ***Fig.2b***.

**b)** Quantification for the TUJ1 fluorescence intensity normalized by DAPI fluorescence intensity in ***Fig.2c***.

**c)** Quantification of pTau217–DAPI colocalization in ***Fig.2b***. Bar graph shows the percentage of DAPI-positive nuclei exhibiting pTau217 signal.

**d)** Quantification of MC1–DAPI colocalization in ***Fig.2c***. Bar graph shows the percentage of DAPI-positive nuclei exhibiting MC1 signal. *N*=5.

**Supplementary Material-7**


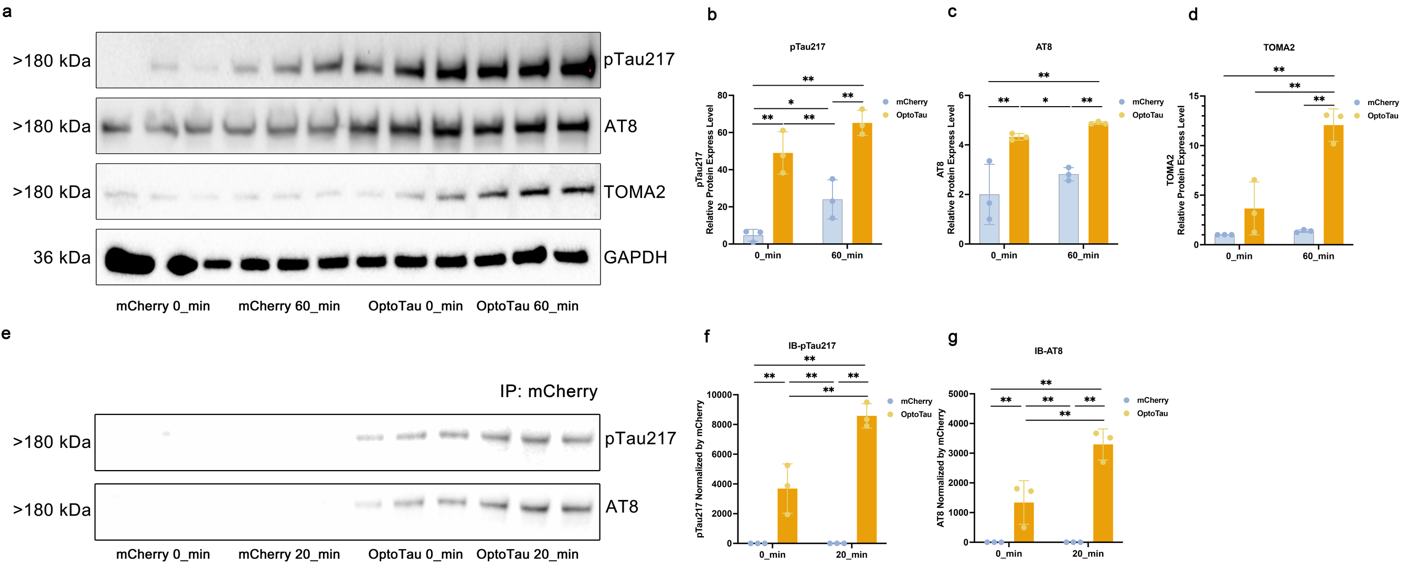


**Supplemental Fig. 5. Tau phosphorylation induced by optogenetic oligomerization in human induced iPSCs.**

**a)** Representative Western blot analysis of pTau217, AT8 and TOMA2 in human iPSC-derived neurons across experimental groups. Total cell lysates were collected from iPSC-derived neurons under control and optogenetic induction conditions. GAPDH was used as a loading control.

**b-d)** Quantification of Western blot results shown in ***panel a***. Band intensities were quantified using ImageJ and normalized to the corresponding GAPDH loading control. Data are presented as fold-change relative to the mean of the control group (set to 1.0).

**e)** Immunoprecipitation of the optogenetically-induced tau complex with a 20-minute illumination paradigm. Lysates from iPSC-induced neurons expressing the optogenetic tau construct (fused to mCherry) were subjected to immunoprecipitation by ChromoTek RFP-Trap® Agarose. The presence of pTau217 and AT8 positive tau phosphorylation in the immunoprecipitated complex was assessed by Western blot.

**f-g)** Quantification of IP results shown in ***panel e***. The pTau217 and AT8 enrichment in the mCherry immunoprecipitate. The band intensity for each protein in the co-IP lane was quantified for pulldown efficiency.

**Supplementary Material-8**

**Table S1. Summary of Human Subject Metadata and Neuropathology.** Clinical and pathological information for all human tissue samples, including diagnosis, Braak staging, age-related metrics, disease duration, APOE status, and demographic variables (race and sex).

| **Primary Neuropathologic Diagnosis** | **Braak Stage** | **Age at Onset** | **Age at Death/ Biopsy** | **Duration (years)** | **APOE** | **Race** | **Sex** |
| --- | --- | --- | --- | --- | --- | --- | --- |
| Control | I |  | 59 |  | E2/3 | b | m |
| Control | I |  | 70 |  | E3/3 | b | m |
| Control | I |  | 72 |  | E3/3 | w | m |
| Control | II |  | 78 |  | E3/3 | w | f |
| Control | IV |  | 84 |  | E2/3 | b | f |
| Control | III |  | 91 |  | E3/3 | w | f |
| Control | III |  | 92 |  | E3/3 | w | f |
| Control | II |  | 94 |  | E3/3 | w | m |
| Control/asymptomatic AD | II |  | 64 |  | E4/4 | w | f |
| Control/asymptomatic AD | IV |  | 80 |  | E3/4 | w | m |
| Control/asymptomatic AD | II |  | 81 |  | E3/3 | w | m |
| Control/asymptomatic AD | I |  | 87 |  | E3/4 | w | m |
| Control/asymptomatic AD | III |  | 87 |  | E2/3 | w | f |
| Control/asymptomatic AD | IV |  | 89 |  | E3/3 | w | m |
| Control/asymptomatic AD | IV |  | 91 |  | E3/3 | w | m |
| AD | VI | 49 | 59 | 10 | E3/3 | w | m |
| AD | VI | 59 | 69 | 10 | E3/4 | w | m |
| AD | VI | 53 | 71 | 18 | E3/4 | w | m |
| AD | VI | 65 | 80 | 15 | E3/4 | b | f |
| AD | V | 77 | 83 | 6 | NA | w | f |
| AD | V | 86 | 92 | 6 | E3/4 | w | f |
| AD | V | 82 | 92 | 10 | E3/3 | w | m |
| AD | VI | 80 | 93 | 13 | E3/4 | w | f |

**Supplementary Material-9**

**Table S2. Antibodies and Reagents.** Comprehensive list of antibodies employed for detection. (a) Primary antibodies categorized by target, source, and experimental concentration. (b) Secondary antibodies used for signal visualization, including host species and conjugation details.

**(a) Primary Antibody Information**

| **Name** | **Host** | **Target** | **Dilution Factor** | **Storage** | **Source** | **Catalog** |
| --- | --- | --- | --- | --- | --- | --- |
| LBR Rabbit PolyAb | Rabbit | Lamin B Receptor | 1:500 IHC; 1:1000 ICC | -20°C | Proteintech | 12398-1-AP |
| AT8 Phosphotau Monoclonal Ab | Mouse | Phosphorylated tau | 1:1000 WB; 1:500 IHC; 1:500 ICC | -20°C | Invitrogen | MN1020 |
| GAPDH Mouse McAb | Mouse | GAPDH (Control) | 1:1000 WB | -20°C | Proteintech | 60004-1-Ig |
| Lamin B2 Rabbit mAb | Rabbit | LaminB2 | 1:1000 WB | -20°C | Cell Signaling Tech | E1S1Q |
| Lamin B2 | Mouse | LaminB2 | 1:400 IHC; 1:500 ICC | -20°C | Invitrogen | 33-2100 |
| Tau5 | Mouse | Phosphorylated tau | 1:400 IHC; 1:400 ICC | -20°C | Millipore | MAB361 |
| PTau 217 Polyclonal Ab | Rabbit | Phosphorylated tau | 1:400 IHC; 1:500 ICC | -20°C | Invitrogen | 44-744 |
| TOMA2 | Mouse | Phosphorylated oligomeric tau | 1:500 IHC | -20°C | Collaborator Dr. Rakez Kayed lab |  |
| Tuj1 Beta Tubulin III | Chicken | Neurons | 1:500 ICC | 4°C | Aves | TUJ |
| MAP-2 | Chicken | Neuronal processes | 1:400 IHC | 4°C | Aves | MAP |
| mCherry mAb (16D7) | Rat | mCherry | 1:500 ICC | -20°C | Invitrogen | M11217 |
| DAPI | Stain | Nuclei | 1:5000 IHC; 1:10000 ICC | -20°C | Thermo Fisher Scientific | 62248 |
| MC1 | Mouse | misfolded tau | 1:500 WB; 1:300 IHC | -20°C, -80°C | The Feinstein Institutes for Medical Research | NA |
| PHF-1 | Mouse | anti-tau pS396 (late); insoluble paired helical filaments (PHFs) | 1:500 WB; 1:300 IHC | -20°C, -80°C | The Feinstein Institutes for Medical Research | NA |

**(b) Secondary Antibody Information**

| Name | Host | Type | Dilution Factor | Storage | Source | Catalog |
| --- | --- | --- | --- | --- | --- | --- |
| Goat anti-Rabbit IgG (H+L) | Rabbit | Alexa Fluor 488 | 2mg/mL | 4°C | Invitrogen | A11008 |
| Goat anti-Rabbit IgG (H+L) | Rabbit | Alexa Fluor 594 | 2mg/mL | 4°C | Invitrogen | A11012 |
| Goat anti-Rabbit IgG (H+L) | Rabbit | hRPC | 1:5000 WB | -20°C | Invitrogen | G21234 |
| Goat anti-rat IgG (H+L) | Rat | Alexa Fluor 488 | 2mg/mL | 4°C | Invitrogen | A11006 |
| Goat anti-Rat IgG (H+L) | Rat | Alexa Fluor 594 | 2mg/mL | 4°C | Invitrogen | A11008 |
| Goat anti-rat IgG (H+L) | Rat | hRPC | 1:5000 WB | -20°C | Invitrogen | A10549 |
| Goat anti-Mouse IgG (H+L) | Mouse | Alexa Fluor 405 | 2mg/mL | 4°C | Invitrogen | A48255 |
| Goat anti-Mouse IgG (H+L) | Mouse | Alexa Fluor 488 | 1mg/mL | 4°C | Invitrogen | A28175 |
| Goat anti-Mouse IgG (H+L) | Mouse | Alexa Fluor 594 | 2mg/mL | 4°C | Invitrogen | A11032 |
| Goat anti-Mouse IgG (H+L) | Mouse | hRPC | 1:5000 WB | -20°C | Invitrogen | G21040 |
| Goat anti-Chicken IgG (H+L) | Chicken | Alexa Fluor 488 | 2mg/mL | 4°C | Invitrogen | A11039 |
| Goat anti-Chicken IgG (H+L) | Chicken | Alexa Fluor 594 | 2mg/mL | 4°C | Invitrogen | A11007 |
| Goat anti-Chicken IgG (H+L) | Chicken | Alexa Fluor Plus 647 | 2mg/mL | 4°C | Invitrogen | A32933 |
| Goat anti-Chicken IgY (H+L) | Chicken | hRPC | 1:5000 WB | -20°C | Invitrogen | A16054 |
